# RNA-Binding Proteins in Dinoflagellates

**DOI:** 10.3390/ijms27010462

**Published:** 2026-01-01

**Authors:** Mariia Berdieva, Pavel Safonov, Sergei Skarlato

**Affiliations:** Laboratory of Cytology of Unicellular Organisms, Institute of Cytology of the Russian Academy of Sciences, 194064 Saint Petersburg, Russia; safonov.incras@gmail.com (P.S.); sergei.skarlato@mail.ru (S.S.)

**Keywords:** conserved domains, dinoflagellate, gene expression, regulation, RNA-binding proteins

## Abstract

The described features of dinoflagellate gene expression indicate the predominance of post-transcriptional and translational regulation over transcriptional control. These microorganisms also exhibit extensive RNA editing and distinctive splicing characteristics. This regulatory landscape underscores the central role of RNA-binding proteins in dinoflagellate biology. In this review, we summarize current knowledge on major RNA-binding protein groups identified or bioinformatically annotated in dinoflagellates, including RNA recognition motif domain-containing proteins, Sm and Sm-like family, KH domain-containing proteins, zinc-finger proteins, and Pumilio family proteins, S1 domain-containing and cold shock domain-containing proteins, DEAD/DEAH-box RNA helicases, and pentatricopeptide repeat proteins. We focus on the features of their conserved domains, their functions in eukaryotes, and available data on their presence and putative roles in dinoflagellate cells. Integrating genomic, transcriptomic, and proteomic evidence, and where possible experimental data, we highlight both their overall conservation and potential lineage-specific traits. Our aim is to provide a concise synthesis of current knowledge, identify key uncertainties, and outline promising directions for future research into the evolution and cellular roles of RNA-binding proteins in this ecologically and biologically remarkable group.

## 1. Introduction

Dinoflagellates are a phylogenetically distinct and ecologically important group of unicellular eukaryotes. They contribute significantly to marine primary production, form endosymbioses essential for coral reef ecosystems, and include species responsible for harmful algal blooms that impact marine food webs and human health [1,2,3]. Dinoflagellates possess complex and variable life cycle routes and stages, which contribute to their ubiquitous distribution and adaptive potential [4,5,6].

It is well established that dinoflagellates exhibit a suite of unusual molecular and cellular features, including extremely large genomes, permanently condensed chromosomes with distinct chromatin architecture, extensive gene duplication, gene organization patterns such as tandem arrays, specific features of splicing, secondary metabolite biosynthesis and other processes [7,8,9,10,11,12,13].

A defining characteristic of dinoflagellate gene expression is the predominance of post-transcriptional and translational regulation over transcriptional control (reviewed in [13]). Although dinoflagellates do demonstrate transcriptional responses to external or internal cues, numerous studies show that mRNA levels often remain stable even when corresponding protein levels oscillate across the diel cycle or under stress. This indicates that post-transcriptional and translational regulation serves as the primary control mechanisms. Such patterns have been documented, for example, for components of the bioluminescence system, metabolic pathways, and cell cycle control machinery [14,15,16,17,18]. Moreover, certain dinoflagellate proteins have been shown to be regulated at the post-translational level via phosphorylation [19].

Several features of dinoflagellate cellular and molecular biology are thought to influence their transcription dynamics, likely contributing to the predominance of post-transcriptional regulation of gene expression. Dinoflagellate chromosomes remain condensed throughout the cell cycle and exhibit a 1:10 protein-to-DNA ratio (in contrast with the more typical equimolar ratio observed in other eukaryotes). Moreover, dinoflagellates encode fewer components of RNA polymerase II compared to classical eukaryotic models, and sequence-specific transcriptional factors in dinoflagellates are known to be scarce [13,20,21].

The trans-splicing phenomenon also occupies a special place in dinoflagellate gene expression. In this process, a short RNA fragment from a small noncoding RNA (the splice leader RNA) is transplanted to the 5′ end of independently transcribed pre-mRNAs to yield mature mRNAs [22]. This mechanism is ubiquitous in dinoflagellates, and most, if not all, of their transcripts appear to undergo post-transcriptional 5′ end modification through trans-splicing (see for review [9,13]).

Dinoflagellates also show widespread RNA editing. This is true for organellar genomes: extensive RNA editing occurs in chloroplasts, where it is essential for the proper folding and function of photosystem proteins [23], and nucleotide modifications are likewise actively employed in mitochondria, potentially contributing to mitochondrial translation efficiency [24,25]. mRNA editing is also observed in nuclear-encoded genes [26,27]. Moreover, most genes appear to be regulated predominantly through one of the post-transcriptional pathways—either RNA editing or alternative splicing—whereas a subset of genes associated with photosynthesis and stress response employs both modes [26]. These observations raise questions regarding the diversity and functioning of the molecular machinery that enables such processes in dinoflagellates. In addition, chromatin-enriched and nuclear proteome analyses reveal an overrepresentation of RNA-binding proteins relative to DNA-binding ones, suggesting an RNA-centric nuclear regulatory architecture [28].

This regulatory landscape places RNA metabolism, and therefore RNA-binding proteins (RBPs), at the core of gene expression control pathways in dinoflagellates. RBPs are typically thought of as proteins that bind RNA targets (single- or double-stranded) through one or multiple globular RNA-binding domains (RBDs) and modulate the fate or function of the bound RNAs [29]. Many RBDs possess modular structure, consisting of multiple repeats of a few basic domains or of several cooperating units [30]. Such an architecture provides advantages in versatility, increased specificity and affinity of binding, and effective regulation of domains or proteins activity [30].

Comparative genomic and transcriptomic studies specify that some RBP families in dinoflagellates exist as large sets of paralogues, in some cases more numerous than in other eukaryotic lineages (e.g., [6,31]). Some RBP-coding genes are arranged in tandem arrays, consistent with the well-documented tendency of dinoflagellate genomes toward extensive duplication and tandem expansion [26,32,33]. The functional significance of these expansions remains unclear but may relate to lineage-specific adaptation and functional diversification—possibilities that are particularly intriguing given the important role of RBPs in post-transcriptional control.

Taken together, these observations highlight RBPs as a functionally crucial protein group in dinoflagellates and suggest that RNA metabolism may represent an influential and even dominant regulatory system in their cells. Large-scale sequencing initiatives, as well as multiple high-quality dinoflagellate genome assemblies, have substantially expanded the catalog of predicted RBPs in this lineage. However, functional studies of specific proteins remain rare. For many families, only domain-level predictions are available, with little biochemical, cellular, or genetic characterization, underscoring a major gap between rapidly accumulating sequence data and mechanistic understanding.

In this review, we provide a concise overview of major RBP families, including features of their conserved domains and canonical roles in eukaryotic cells, and summarize all available evidence for their presence and function in dinoflagellates, drawing on transcriptomic and genomic surveys, nuclear proteomics, and, where possible, experimental studies. In some cases, we also conduct additional sequence-level analyses to supplement or clarify existing data. For several RBP families, sufficient evidence allows meaningful conclusions, whereas for others only basic information about the presence of homologues is available. Our aim is to present an accurate and restrained summary of current knowledge, highlight key uncertainties, and outline promising directions for future research to bridge the gap between sequence-based predictions and cellular functions of RBPs in these biologically and ecologically remarkable organisms.

## 2. RNA-Binding Proteins Review

In the following sections, we examine the principal classes of RNA-binding proteins, encompassing RNA recognition motif-containing proteins, Sm and Sm-like proteins, KH domain-containing and zinc-finger proteins, Pumilio family proteins, oligosaccharide-/oligonucleotide-binding fold protein group including S1 domain-containing and cold-shock domain-containing proteins, DEAD/DEAH-box RNA helicases, and pentatricopeptide repeat proteins. For each group, we first outline the defining features of their conserved domains—covering amino acid composition, fold architecture, and principles of RNA binding and activity—and summarize their canonical functions in eukaryotic cells. We then compile the evidence currently available for dinoflagellates, including their presence and diversity, structural characteristics, and potential cellular roles. Table 1 provides a concise synthesis of the major groups considered here, highlighting their characteristic domain architectures and representative folds, broad cellular functions, and the present state of knowledge for dinoflagellates (ranging from evidence of presence based on genomic, transcriptomic, and/or proteomic data, to differential gene expression analyses, sequence and structural characterization, and experimental studies).

## 3. RNA Recognition Motif Domain-Containing Proteins

RNA recognition motif (RRM) domain-containing proteins are found in all major branches of life, including viruses. However, they are especially abundant in eukaryotes, where the RRM is the most common RBD [79,80,81,82]. In eukaryotes, RRM-containing proteins participate in all major post-transcriptional processes, including pre-mRNA processing, splicing, RNA stabilization, and editing [79,83,84].

**Figure 1:**
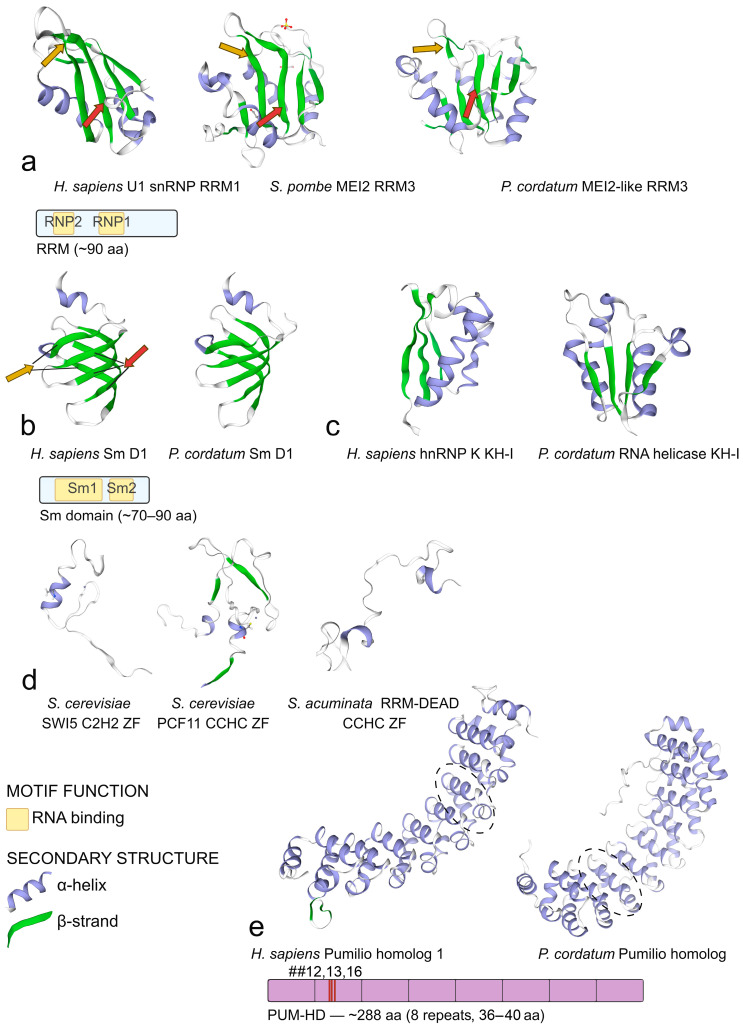

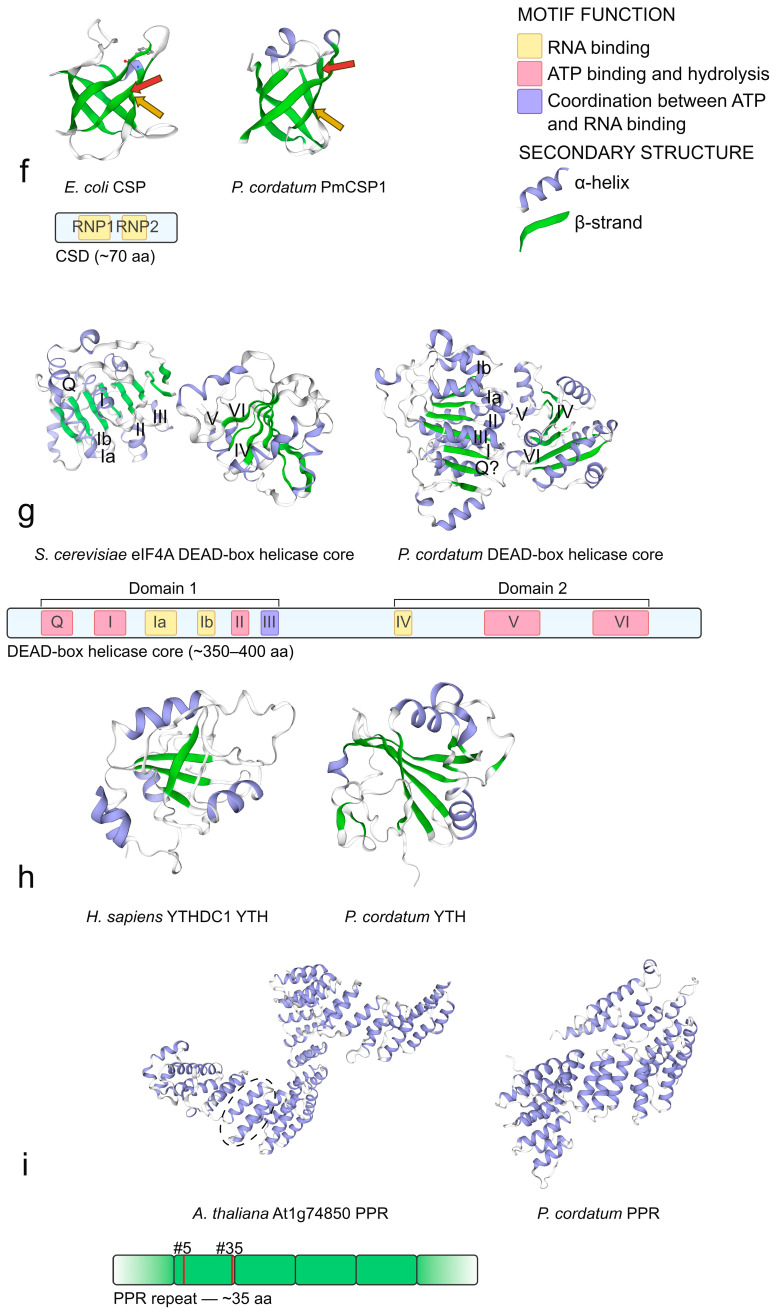
Tertiary structure models of conserved domains and general domain organization of RNA-binding proteins considered in this review. (**a**) RNA recognition motif (RRM) domain (RRM1) from *Homo sapiens* U1 small nuclear ribonucleoprotein 70 kDa (from model AF-P08621-2-F1-v6), RRM3 from *Schizosaccharomyces pombe* MEI2 protein (PDB code 7eio), and RRM3 from *Prorocentrum cordatum* MEI2-like homolog CAK0832983.1 (AlphaFold Server https://alphafoldserver.com/ (accessed on 12 December 2025) prediction, pTM = 0.85). (**b**) Sm domain from *H. sapiens* small nuclear ribonucleoprotein Sm D1 (from model AF-P62314-F1-v6) and *P. cordatum* putative small nuclear ribonucleoprotein Sm D1 CAK0795168.1 (AlphaFold Server prediction, pTM = 0.62). (**c**) type I KH domain from *Homo sapiens* heterogeneous nuclear ribonucleoprotein K (PDB code 1zzk) and *P. cordatum* CAK0809374.1 putative KH-containing RNA helicase (AlphaFold Server prediction, pTM = 0.73). (**d**) Zinc-finger domains: C2H2 type from *Saccharomyces cerevisiae* factor SWI5 (PDB code 1ncs), CCHC type from *S. cerevisiae* component of the cleavage factor PCF11 (PDB code 2nax), and CCHC type in modification CX_2_CX_3_HX_4_C from *Scrippsiella acuminata* RRM-DEAD helicase 20130930|942_1 (AlphaFold Server prediction, pTM = 0.37). (**e**) Pumilio domain from *H. sapiens* Pumilio homolog 1 (PDB code 1ib2) and *P. cordatum* Pumilio homolog CAK0819378.1 (AlphaFold Server prediction, pTM = 0.89). (**f**) Cold shock domain from *Escherichia coli* CSP (PDB code 8rxe) and *P. cordatum* PmCSP1 CSD-containing protein QYF11783.1 (AlphaFold Server prediction, pTM = 0.64). (**g**) DEAD-box helicase core from *S. cerevisiae* translation initiation factor 4A eIF4A (PDB code 1fuu) and from *P. cordatum* DEAD-box helicase CAK0847872.1 (AlphaFold Server prediction, pTM = 0.86). In the *P. cordatum* protein model, the region predicted to correspond to the Q domain is indicated by “Q?”. (**h**) YTH domain from *H. sapiens* (PDB code 6we8) and *P. cordatum* YTH-containing protein CAK0851154.1 (AlphaFold Server prediction, pTM = 0.75). (**i**) pentatricopeptide repeat proteins—*Zea mays* PPR10 (PDB code 4m57) and *P. cordatum* PPR protein CAK0882574.1 (AlphaFold Server prediction, pTM = 0.59). In Pumilio and PPR proteins, a single structural unit composed of one repeat is indicated by a dashed oval. For RRM (**a**), Sm domain (**b**), CSD (**f**), and DEAD-box helicase core (**g**), generalized domain organization schemes are shown. The block color reflects the functional role of key motifs; red arrow indicates motif 1 in the domain (RNP1 in RRM and CSD, Sm1 in Sm domain), sand arrow indicates motif 2 in the domain (RNP2 in RRM and CSD, Sm2 in Sm domain). The generalized organization of Pumilio and PPR proteins, which consist of multiple tandem repeats, is also shown (**e**,**i**), with the positions of RNA-binding residues indicated by red lines.

A single RRM domain is about 90 amino acids long and contains two conserved ribonucleoprotein (RNP) motifs essential for binding ssRNA: RNP1 ([KR]G[FY][GA][FY][VIL]X[FY]) and RNP2 ([IVL][FY][IVL]XNL) [85,86] (Figure 1a). Structurally, RRM comprises both β-strands and α-helices, arranged in a characteristic β_1_α_1_β_2_β_3_α_2_β_4_ fold, with the central β-strands bearing the two RNP motifs [87,88] (Figure 1a). RNA recognition therefore occurs on the surface of the β-sheet. Three conserved residues are the most crucial for binding: Arg/Lys binds the RNA backbone, while two aromatic residues interact with bases. Additional secondary structures outside the two conserved motifs can also interact with RNA, increasing the specificity of recognition (reviewed in [30]).

RRM-containing proteins are abundant in dinoflagellates. On the proteomic level, a study of the chromatin-associated proteins in *Lingulaulax polyedra* (syn. *Lingulodinum polyedra*; in this review, we follow currently accepted species names according to the AlgaeBase https://www.algaebase.org/ (accessed on 19 November 2025) and WoRMS https://www.marinespecies.org/ (accessed on 19 November 2025) databases, providing synonyms in parentheses upon first mention if they were used in the original publications) detected 56 proteins harboring RRMs. Of these, 44 contained only RRM domains—either one, two, or three—while the remainder also carried other domains [28]. Simultaneously, several groups of RRM-containing proteins have been bioinformatically annotated in dinoflagellates at the genomic and transcriptomic levels. Considering the diversity of RRM-containing proteins, below we briefly characterize each of those evolutionary and/or functional groups separately.

### 3.1. MEI2-like Proteins

MEI2 is an RNA-binding protein originally described in fission yeast *Schizosaccharomyces pombe*, where it was shown to be a key regulator of transition from mitosis to meiosis [89]. In general, it binds and stabilizes long non-coding meiRNA that serves as a sponge for Mmi1 (meiotic mRNA interceptor 1) protein during meiotic initiation; besides, it sequesters Mmi1 mRNA [90]. Subsequently, homologues of MEI2 were predicted and identified in higher plants [91,92,93] and diverse protists (e.g., [93,94,95]), including dinoflagellates [6,32,34,35,37,39,40]. Structurally, MEI2 contains three RRM domains [94], with the third RRM domain (RRM3) being the most conserved [94]. Moreover, RRM3 possesses a unique architecture as it is extended by an additional C-terminal α-helix followed by two β-strands [94] (Figure 1a). Remarkably, in myzozoans, i.e., all alveolates except ciliates [96], the annotated MEI2-like homologues contain only RRM3, except for two homologues of *Kryptoperidinium triquetrum* (syn. *Kryptoperidinium foliaceum*) that apparently originated from the diatom endosymbiont, and two proteins of *Perkinsus chesapeaki* and *P. olseni* [31,94]. The architecture of dinoflagellate RRM3 appears to be similar to the canonical one (Figure 1a). The study by Palii and co-authors [31] also revealed varying numbers of homologues with the same structure in other eukaryotic groups, indicating that the loss of RRM1 and RRM2 occurred independently in different lineages (Table S1 in [31]; here and in following cases, the sources of the mentioned supplementary data from other works are listed in Table S1). In dinoflagellates, genomic and transcriptomic analyses indicate that the repertoire of MEI2-like proteins expanded during the evolution of the group [6,31]. Early-branching taxa encode relatively few homologues (four in *Amoebophrya* species and about 24 in *Oxyrrhis marina*), whereas the core dinoflagellate *Prorocentrum cordatum* possesses as many as 89 homologues [31].

MEI2 homologues are widely present in dinoflagellate transcriptomes and genomes. However, the absence of direct studies of proteins or loss-of-function phenotypes limits our ability to functionally characterize dinoflagellate MEI2 homologues. Nevertheless, differential gene expression analyses, supplemented with phylogenetic reconstructions, provide important insights into their possible roles in these organisms.

Accumulating transcriptomic evidence links changes in *MEI2*-like gene expression to distinct life-cycle stages and responses to external stressors in dinoflagellates. As discussed previously in [31], two broad transcriptional patterns are emerging. In the present review, we further refined and expanded this distinction and summarized it in Table 2. In the first pattern, certain MEI2-like genes show altered expression during vegetative life-cycle stages, particularly during cyst formation. In cold-stress-induced temporary cysts of *P. cordatum*, five differentially expressed genes (DEGs) showed reduced expression (protein product sequences CAK0884715.1, CAK0887363.1, CAK0909770.1, CAK0844294.1, and CAK0818609.1), with two of them (CAK0884715.1, CAK0818609.1) exhibiting elevated expression during preparation for excystment [36]. Three DEGs (protein products CAK0836878.1, CAK0828848.1, and CAK0884998.1) also differed in expression relative to motile cells [36]. In dark- and cold-induced temporary (pellicular) cysts of *Scrippsiella acuminata* (syn. *Scrippsiella trochoidea*), both up- and down-regulated DEGs encoding MEI2 homologues were annotated in the same dataset [34,38]. Stress-induced coccoid cells in *Effrenium voratum* demonstrated down-regulated *MEI2*-like DEGs [37]. Additionally, phosphate deficiency suppressed MEI2-coding genes [71].

Unlike the expression pattern described for *P. cordatum* in [36], *S. acuminata* cysts showed increased expression of gene coding for SMC3 (structural maintenance of chromosomes protein 3), which may be associated with meiosis [38]. SMC3 is a component of the cohesin complex that is essential for sister chromatid pairing prior to division [97]; nevertheless, this complex mediates cohesion in both mitosis and meiosis [98].

Given the rapid return of *S. acuminata* cysts to motility under favorable conditions [38], together with the *P. cordatum* data [36], some MEI2 homologues may play a role in excystment. Up- and down-regulated *MEI2*-like genes have also been reported in *S. acuminata* resting cysts [34]. Notably, *SPO11* and *DMC1* genes, both listed in the meiosis detection toolkit [99], were also up-regulated. This may indicate recombination events in *S. acuminata* cysts coupled with sexual process. However, because mature resting cysts were sampled for RNA sequencing [34], preparation for excystment could also have occurred in this case.

In the second pattern, regulation of *MEI2*-like genes coincides with the regulation of a larger number of meiosis-specific genes, which more confidently indicates the preparation for meiosis. In *Alexandrium minutum* under nitrogen deficiency, 47 GO terms related to meiosis were enriched, strongly suggesting the induction of sexuality. Among these, a *MEI2* homologue was up-regulated [40]. Moreover, active meiotic processes during bloom events have been documented in several dinoflagellate species [6,39]. The concomitant up-regulation of at least some genes encoding MEI2 homologues is consistent with the proposed role of MEI2 as a regulator of meiosis. Nevertheless, given the large number of *MEI2*-like genes showing altered expression (see Figure S2 in [6]), we cannot exclude that a subset of these genes may belong to another functional group of MEI2-like proteins and may instead be involved, for example, in rapid post-transcriptional regulation of gene expression during bloom development under natural conditions.

Thus, dinoflagellates potentially possess at least two functional sets of MEI2 homologues corresponding to the above activity patterns. Importantly, it is not surprising that some MEI2-like proteins could have functions beyond meiosis regulation. For example, in plants, MEI2 homologues were shown to be expressed in vegetative tissues [100], where some of them are involved in control of leaf initiation [92,93,100]. Furthermore, phylogenetic analysis of a large dataset of dinoflagellate MEI2-like proteins revealed at least six distinct clades of homologues [31]. Notably, the MEI2 homologues implicated in the formation of *P. cordatum* temporary cysts clustered within two of these clades [36]. These results indicate that possibly even more pools of MEI2 homologues coexist in dinoflagellates.

### 3.2. hnRNP Proteins, snRNP Proteins and SR Proteins

The hnRNP proteins constitute the protein component of heterogeneous nuclear ribonucleoproteins (hnRNPs). These RNA-binding proteins associate with nascent transcripts and influence splicing, mRNA export, stability, localization, and translation (reviewed in [79]). Most of hnRNP proteins contain one or several RRM domains [79]; for example, members of the hnRNP A/B family possess two N-terminal RRMs (reviewed in [101]).

The small nuclear ribonucleoproteins (snRNPs) are core components of the spliceosome. Each snRNP is composed of a specific small nuclear RNA (snRNA), a common set of RNA-binding Sm proteins (considered separately below), and a set of auxiliary proteins, that are specific to each particle (reviewed in [102,103]). Several of these auxiliary proteins carry RRM domains, which bind the corresponding snRNA molecules (e.g., [104,105,106]).

SR proteins represent a family of RNA-binding factors involved in the control of both constitutive and alternative splicing [107,108]. Structurally, SR proteins contain at least one RRM domain and an Arg-Ser–rich region known as the RS domain; some family members also contain a second RRM or additional auxiliary domains [107,108]. Strong evidence supports the antiquity of this family: phylogenetic reconstruction of SR protein RRMs from a plethora of diverse organisms indicates that the diversification of their main structural types predates the divergence of the major eukaryotic lineages [80]. Notably, the analysis by Califice et al. [80] did not include dinoflagellate SR proteins, although it did feature diverse homologues from apicomplexans.

In dinoflagellates, cis-splicing (“canonical”, i.e., intramolecular splicing within a single pre-mRNA molecule) is generally thought to be less prominent than in many other eukaryotes given the paucity of introns in their genes. Nonetheless, both snRNAs and some of snRNP proteins were shown to be highly conserved between mammals and dinoflagellates (reviewed in [20,21]). At least some RRM-containing auxiliary snRNP proteins are conserved across metazoans, yeasts and plants, suggesting their evolutionary antiquity [108]. Indeed, a brief blastp search against the NCBI ClusteredNR database using human U1-70K protein (NP_001287998.1) as a query revealed strongly-supported homologues in both basal and core dinoflagellates, e.g., CAD7962409.1 from *Amoebophrya* sp. A25 (E-value = 6 × 10^−52^), and CAE7225710.1 from *Symbiodinium pilosum* (E-value = 4 × 10^−51^; here and further, the list of the revealed homologues is non-exhaustive). Subsequent analysis utilizing the CD-search tool (https://www.ncbi.nlm.nih.gov/Structure/cdd/wrpsb.cgi, accessed on 12 December 2025) revealed U1-70K-specific N-terminal domain (U1snRNP70_N), followed by an RRM domain in both homologues (Figure S1a, multiple sequence alignment was generated using MAFFT7 [109] implemented in the Unipro UGENE software package [110]).

Furthermore, nearly all nuclear-encoded mRNAs in dinoflagellates undergo trans-splicing, in which an exon derived from a separately transcribed splice leader (SL) RNA is joined to the 5′ end of an mRNA during its maturation (reviewed in [13,20]). Alternative splicing, previously thought to be absent in dinoflagellates [20], has since been confirmed by genomic analyses (reviewed in [10]). Taken together, these points indicate that the repertoire of dinoflagellate hnRNP, snRNP, and SR proteins warrants detailed investigation. A blastp search against the NCBI ClusteredNR database using human SRSF2 protein (NP_001182356.1) as a query retrieved a number of dinoflagellate homologues, e.g., CAE8617636.1 (E-value = 4 × 10^−22^), and CAJ1443725.1 (E-value = 7 × 10^−21^). For these sequences, the CD-search tool predicted an N-terminal RRM domain (Figure S1b). Although the algorithm did not predict the RS domain in the retrieved homologues, it also failed to detect this domain in the query sequence. Nevertheless, an Arg–Ser-rich region can be clearly distinguished in the multiple sequence alignment (Figure S1b). Notably, some of the homologues are annotated correspondingly as SR proteins (e.g., CAE6927798.1 and CAE7359325.1 of *Symbiodinium* sp. CCMP2592).

### 3.3. RRM-Containing Translation Initiation Factors

In contrast to translation initiation in bacteria, which involves only three factors (IF1–3) [111], the eukaryotic initiation machinery includes a much larger set of proteins and multisubunit complexes [112], several of which harbour RRM domains. Among them is eIF4B—a protein mediating the association between the small (40S) ribosomal subunit and mRNA [113,114]. Besides an N-terminal RRM domain that binds 18S rRNA, eIF4B also contains a second C-terminal RBD, termed the basic domain (BD), which binds mRNA [115,116].

The eIF3 complex, essential for binding the 40S subunit, mRNA and other initiation factors, comprises five core proteins in yeasts, whereas mammals possess a larger set in which these five core subunits are supplemented with seven additional ones, for a total of twelve [117,118]. Two of the core subunits—eIF3b and eIF3g—contain RRMs [119,120,121,122]. eIF3b participates in the nucleation of the entire eIF3 complex, promoting its stabilization on the 40S ribosomal subunit through interaction with 18S rRNA. Notably, the RRM domain of eIF3b also mediates protein–protein contacts with other eIF3 subunits [118,122,123]. eIF3g, by means of its RRM domain, contributes to linear scanning of the mRNA 5′ UTR for the start codon and may also mediate reinitiation following translation termination on short upstream ORF [124]. In addition, the RRM of eIF3g participates in 18S rRNA binding [125,126].

Poly(A)-binding proteins (PABPs), although not formally classified as initiation factors, also contain RRM domains and, through interaction with eIF4G, mediate mRNA circularization, thereby promoting recruitment of the 40S ribosomal subunit. Besides translation initiation, PABPs participate in mRNA polyadenylation, nuclear export, and degradation. Canonically, cytoplasmic PABPs bear four RRM domains, and some also contain a C-terminal PABP-specific domain MLLE, whereas nuclear PABPs typically have only a single RRM and lack the MLLE domain (reviewed in [127,128]). Earlier reviews (e.g., [127]) noted that, while some metazoans and plants encode multiple PABP genes, unicellular eukaryotes (except yeasts) typically possess a single homologue. However, subsequent research has shown that multiple PABPs in single-celled organisms may be more common. In *Leishmania major*, three PABP paralogues have been identified, and *Trypanosoma* species possess at least two; all identified trypanosomatid PABPs are characterized by a four-RRM + C-terminal MLLE architecture [129]. More recently, two PABPs were identified in *Plasmodium yoelii*: one with a four-RRM + C-terminal MLLE architecture and another bearing only a single RRM [130].

In general, dinoflagellates possess a largely canonical repertoire of eukaryotic initiation factors [13]. Nevertheless, certain factors, including RRM-containing ones, appear to be missing. A notable example is eIF4B, which has not been identified or predicted in dinoflagellates [13]. In *L. polyedra*, four of the five core subunits of the eIF3 complex—a, b, d, and e—have been identified at the proteomic level [131], i.e., eIF3g has not been detected. As for the diversity of dinoflagellate PABPs, no direct information is currently available in the literature. Although proteins annotated as PABPs have been listed in genomic datasets (e.g., [26]), such general annotations do not allow the domain composition to be inferred. We performed a blastp search against the NCBI ClusteredNR database using the human PABPC1 protein (NP_002559) as a query, and it yielded several dinoflagellate homologues that we considered *bona fide* PABPs (E-value < 3 × 10^−90^ for all). According to conserved domain predictions, these sequences contain both RRM domains (RRM1–4, except for CAE8617444.1 from *Polarella glacialis* CCMP1383, which contains only RRM2–4) and a C-terminal PABP-specific domain (Figure S1c). At least three homologues were identified in *P. glacialis* CCMP1383 (CAE8641676.1, CAE8622414.1, CAE8617444.1), two in *P. glacialis* CCMP2088 (CAE8685442.1, CAE8738554.1), and two in *P. cordatum* (CAK0907260.1, CAK0870724.1) proteomic datasets corresponding to genome assemblies GCA_905237085.1, GCA_905237095.1, and GCA_963575745.1, respectively [26]. These results indicate the likely coexistence of multiple paralogues in at least some dinoflagellate species.

## 4. Sm and Sm-like (Lsm) Proteins

Sm and Sm-like (Lsm) proteins represent an evolutionarily conserved family of small RNA-binding proteins ubiquitously found in bacteria, archaea, and eukaryotes [132,133]. In both eukaryotes and prokaryotes, each (L)Sm monomer folds into a characteristic structure consisting of N-terminal α-helix followed by five strongly bent antiparallel β-strands (β1–β5) [134] (Figure 1b). Canonical proteins assemble into multimeric ring structures: eukaryotic Sm proteins form a heptameric ring [135], whereas Lsm proteins typically form heteromers or homomers of six or seven subunits [136]. Sm and Lsm complexes preferentially bind uridine-rich single-stranded 3′ ends. RNA oligonucleotides generally bind inside the central lumen of the ring, one nucleotide per subunit (monomer) (see for review [137]).

The heptameric Sm ring consists of the core Sm proteins (subunits) of subtypes B (or alternatively B’ or N), E, F, G, D1, D2, and D3, which serve as its structural elements. This heteromeric complex is localized in the nucleus and binds spliceosomal U1, U2, U4, and U5 snRNAs, thereby constituting snRNPs, which in turn serve as building blocks of the spliceosome [136]. Notably, Sm proteins can be involved not only in cis-splicing but also in trans-splicing, a process that occurs in dinoflagellates. This is consistent with the fact that SL RNAs share similarities with spliceosomal snRNAs, including a U-rich Sm-protein-binding site [138].

The nuclear Lsm2–8 complex can act as a chaperone that interacts with U6 snRNA and promotes the reassociation of the U4 and U6 snRNPs after splicing. In contrast, the cytoplasmic Lsm1–7 complex recognizes oligoadenylated mRNAs and protects 3′ end of the mRNA from exonucleases, while also recruiting the decapping machinery and promoting mRNA decay (see for review [133]).

Sm proteins share a conserved bipartite Sm domain composed of two sequence motifs, Sm1 and Sm2, separated by a variable linker [139,140]. This domain is followed by a glycine- and proline-rich C-terminal tail [140]. The Sm1 motif typically lies in the first three β-strands, while Sm2 covers strands four and five [133,141]. Analyses of eukaryotic Sm protein sequences reveal several highly conserved residues in Sm1. In particular, these include a Gly residue in β1, which contributes to inter-subunit contacts, an Asp residue that is important for proper structural orientation, and an Asn, the third of the three key RNA-binding residues located in the loop between β2 and β3 [139,140,141]. In Sm2, the conserved region corresponds to the loop between β4 and β5, where adjacent Arg (or Lys, as in Sm-E) and Gly (or Cys, as in Sm-F) residues are typically found—the former participating in RNA binding and the latter contributing to structural stability. A nonpolar hydrophobic region precedes them in β4 [139,140,141].

Sm proteins appear to be well-conserved in dinoflagellates and largely similar to canonical ones (Figure 1b), despite the distinctive features of dinoflagellate splicing. Random sequencing of SL-based cDNA libraries revealed several sequences encoding Sm proteins highly similar to canonical Sm ring components in *Pfiesteria piscicida* and *Karlodinium veneficum* (syn. *Karlodinium micrum*) [22]. Lsm domain-containing sequences were also listed among components of the splicing machinery inventoried in *Pyrocystis lunula* [46]. Although not explicitly discussed, Lsm domains or Lsm-coding genes are annotated in dinoflagellates from diverse lineages, including basal groups (e.g., [13,26,33,43,44,45]). Sm ring components can also be found in various datasets: for example, all core Sm proteins B, F, E, G, D1, D2, and D3 are present in the *P. glacialis* reference proteome UP000654075 in UniProtKB database (https://www.uniprot.org/, accessed on 12 December 2025), and nearly all components—except G subunit—are annotated in *P. cordatum* genome assembly [26]. Random testing of Sm domains confirmed a high level of conservation in dinoflagellate sequences, reflected in their close similarity to canonical eukaryotic Sm proteins and the strong preservation of residues essential for Sm function.

Other lines of evidence also support the conservation of Sm proteins in dinoflagellates. Reddy et al. [41] demonstrated the presence of U1–U6 snRNAs and Sm proteins associated with them in *Crypthecodinium cohnii* using human anti-Sm antibodies. These antibodies recognize the so-called “Smith antigen”, i.e., the complex of Sm proteins bound to spliceosomal snRNAs [142]. Alverca et al. [42] used serum recognizing symmetrical dimethylarginine to label nuclear proteins, including Sm proteins, in several dinoflagellate species. Symmetrical dimethylarginine is a post-translational modification of an arginine side chain found in C-terminal tail in some core spliceosomal Sm and Lsm proteins [143], though it also occurs in other nuclear proteins such as hnRNPs and coilin [144,145]. The authors attributed labeling of protein bands of the expected molecular mass to Sm proteins, although at the level of immunofluorescence and immunoelectron microscopy this interpretation remained tentative [42]. In the nuclei of *Amphidinium carterae*, *Prorocentrum micans*, *Akashiwo sanguinea*, *Alexandrium fundyense* (syn. *Alexandrium catenella*), and *Alexandrium minutum* (syn. *Alexandrium lusitanicum*), they observed perichromosomal labeling related to perichromosomal granules enriched in splicing factors, as well as labeling in Cajal-like bodies [42].

Considering conservation of the Sm complex, it should be noted that the Sm-binding site in SL RNA appears to exhibit distinctive features in dinoflagellates. According to data obtained by Zhang et al. [22,48], SL RNAs from several species (*Karenia brevis*, *P. piscicida*, *K. veneficum*, *P. cordatum*, *P. glacialis*, *Heterocapsa arctica*), contain a conserved Sm binding motif AUUUUGG (AUGUUGG in one SL RNA type in *P. glacialis*), located within a stem-loop structure in the exon region of the 50–60 bp SL RNA that is ultimately trans-spliced to mRNAs. In other trans-splicing organisms, by contrast, the Sm site is located in the intron. Because the Sm ring functions as part of the nuclear import signal, its retention on the mature mRNA could potentially interfere with cytoplasmic targeting [22]. A different study [47], questioned this exon-based localization in *K. brevis* and identified a U-rich AGGCCUCUUUUG region downstream of the exon in the 150-bp SL RNA. More recently, Song et al. [49] described a U-rich motif GUUUUC in introns of SL genes in *Fugacium kawagutii* (syn. *Symbiodinium kawagutii*) as a candidate unusual Sm-binding site in SL RNAs of 103–292 bp. Further research is needed not only to resolve discrepancies among reported SL RNA sequences and possible diversity in Sm-binding sites, but also to clarify Sm-complex assembly and dynamics during mRNA maturation, ultimately shedding light on the unique features of splicing in dinoflagellates.

## 5. KH Domain-Containing Proteins

First identified in the human heterogeneous nuclear ribonucleoprotein K (hnRNP K), the K homology (KH) domain is present in proteins across bacteria, archaea, and eukaryotes, often occurring in tandem copies. In general, eukaryotic proteins tend to contain more KH domains than prokaryotic proteins, although some eukaryotic KH proteins bear only a single domain [146].

KH domains are approximately 70 amino acids long and can adopt two topologies that share the same β_1_α_1_α_2_β_2_ core (Figure 1c). Eukaryotic proteins usually possess type I domains with a β_1_α_1_α_2_β_2_β′α′ architecture, while prokaryotic proteins typically bear domains that adopt a β′α′β_1_α_1_α_2_β_2_ fold (type II KH). In type I KH domains, the β-strands form a β_1_β′β_2_ sheet in which β′ is antiparallel to β_1_ and β_2_ [146]. A short loop connecting the α_1_ and α_2_ helices contains a GXXG motif that is crucial for RNA binding: proteins with the typical KH fold but modified sequence of this motif do not exhibit RNA-binding activity [147]. The binding of the nucleic acid backbone occurs in a cleft formed by the two α-helices and the β-sheet. Typically, an individual KH domain binds four nucleotides, and the specificity of recognition increases when multiple KH domains are present within a single protein [146,147]. KH domain-containing proteins are involved in the regulation of transcription, mRNA processing, and translation [79]. In both prokaryotes and eukaryotes, some of them have been shown to be membrane-associated [148].

Together with RRM and zinc-finger domains, KH domains are among the most common RBDs [82]. Consequently, it is not surprising that dinoflagellates appear to possess a significant diversity of KH domain-containing proteins (see an example of the predicted tertiary structure in Figure 1c). At the transcriptomic level, such proteins have been annotated in basal parasitic dinoflagellates *Amoebophrya* sp. and *Hematodinium* sp. [13,43]. KH domain-containing proteins or KH domains were also predicted in genomes and/or transcriptomes of several core dinoflagellates, including *Symbiodinium microadriaticum*, *S. natans*, *S. tridacnidorum*, *Breviolum minutum* (syn. *Symbiodinium minutum*), *F. kawagutii*, *L. polyedra*, *C. cohnii*, *Durinskia* sp., *Pyrocystis lunula*, and *P. cordatum* [13,26,44,46,50,51]. At the proteomic level, KH domains were identified among chromatin-associated proteins of *L. polyedra*: the study by Beauchemin and Morse [28] identified 34 proteins harbouring 1–6 KH domains, of which 28 possessed only them.

## 6. Zinc-Finger Proteins

Zinc-finger proteins are among the most widespread nucleic acid-binding factors found across all three domains of life, with their greatest diversification occurring in eukaryotes [149,150,151]. They bear compact (about 30 amino acids long), Zn^2+^-stabilized domains (ZF) that differ in the number and arrangement of zinc-coordinating cysteines and histidines, as well as in their preferred interaction partners [150,152].

Types of ZF domains are designated according to the composition of conserved amino acids that bind zinc ions. The most abundant eukaryotic ZF is characterized by a CX_4_CX_12_HX_3_H motif, hence the designation C2H2. These domains function primarily as DNA-binding units, although numerous C2H2-containing proteins have been shown to interact with RNA [150,153,154,155,156]. In contrast to the C2H2 ZFs, the CCCH, CCHC, and CCCC subtypes are known primarily as RBDs found in proteins involved in RNA folding, processing, and turnover [153,157,158,159,160,161,162].

The specificity of RNA recognition and the mechanism of binding differ among ZF types. C2H2 domains adopt a ββα fold and recognize RNA mainly through side chains of residues on the α-helix (Figure 1d). At least two modes of recognition are known: in transcription factor IIIA (TFIIIA) of *Xenopus* oocytes, different C2H2 domains bind either the backbone or individual bases of the 5S rRNA molecule [163]. CCCH domains are compact structures composed of short α-helices and 3_10_-helices [164] and either bind specific RNA sequences or secondary structures [153,160]. In CCCH-containing protein tristetraprolin (TTP), the recognition of AU-rich elements in 3′ UTRs of mRNAs is achieved through interaction of protein main chain with RNA bases [153]. The CCHC domain is composed of two short β-strands, connected by a specific turn called “zinc knuckle”, and a short α-helix [162] (Figure 1d). These domains recognize stem-loop structures in either ssDNA or RNA by interacting with both bases in the loop (facilitating sequence-specific recognition of, for example, G-rich sequences) and the backbone [162,165].

As in the case of the KH domain-containing proteins, zinc-finger proteins bearing C2H2, CCCH and CCHC types of domains were listed both in genomic/transcriptomic and proteomic datasets of dinoflagellates (e.g., [13,26,28,43,44,50,51])

## 7. Pumilio Family Proteins

The Pumilio RNA-binding protein family, or the PUF (Pumilio and Fem-3 binding factor) family, comprises highly conserved eukaryotic proteins [166] that contain the sequence-specific RNA-binding Pumilio homology domain (PUM-HD), or Puf domain (reviewed in [167,168]). The PUM-HD consists of eight tandem copies of repeated motif of 36–40 amino acids and N- and C-terminal flanking regions [167,169,170]. Each repeat folds into three α-helices arranged in a triangular configuration; together, the repeats pack into a right-handed superhelix curved in the shape of half a doughnut [170] (Figure 1e). RNA is bound in a sequence-specific manner on the concave surface of the superhelix, with each repeat recognizing a single RNA base through three amino acid side chains at conserved positions 12, 13, and 16 [171]. The type of these residues depends on the position and repeat number. In most cases, PUF proteins bind specific sites in the 3′ untranslated regions of target mRNAs and also can recruit other protein machineries; they mediate post-transcriptional control of gene expression, either by promoting or repressing translation and by regulating mRNA stability [168]. They have been shown to regulate a wide range of processes across eukaryotes, including stem cell control, developmental patterning and gametogenesis, organelle biogenesis, and stress response [168].

Dinoflagellates appear to possess repertoire of PUF proteins. The PUF-coding genes have been found in transcriptomic data from early-branching parasitic dinoflagellates *Amoebophrya* sp. [43] and *Hematodinium* sp. [13]. Among the core groups, PUM-HDs (domain pfam00806) were inventoried in genomes and/or transcriptomes of Symbiodiniaceae species from various clades (*S. microadriaticum*, *B. minutum*, *F. kawagutii*, *Cladocopium goreaui*, *Symbiodinium aenigmaticum*, *S. pseudominutum*, *S. psygmophilum*), as well as in omics data from *K. brevis*, *P. cordatum*, *L. polyedra*, *A. carterae*, *C. cohnii*, *P. lunula*, and *Alexandrium minutum* [13,26,44,46,50,54,55]. Pumilio domain was listed among RNA binding domains detected during the proteomic analysis of the chromatin-enriched samples of *L. polyedra* [28].

We did not find any dedicated studies analyzing the amino acid composition, domain architecture, or folding features of PUM-HD-carrying proteins in dinoflagellates. Nevertheless, to obtain a first approximation of PUM-HD conservation, we selected amino acid sequences containing either a Pumilio-family RNA binding repeat (pfam00806) or Pumilio-family RNA binding domain (cd07920) from the *P. cordatum* genome assembly GCA_963575745.1 proteome dataset [26] (Figure S2). The multiple amino acid sequence alignment confirmed that the regions within the repeats corresponding to the inner surface of the protein are relatively well conserved (Figure S2). Indeed, dinoflagellate Pumilio-containing proteins were among the RNA-binding proteins showing the highest degree of sequence similarity (best E-values in tblastn analysis) to mammalian queries [13]. The architecture of dinoflagellate PUM-HD-carrying proteins also appears to be similar to the canonical one (Figure 1e).

Data on the potential functioning of PUF proteins in dinoflagellate cells remain extremely limited. In *S. microadriaticum* cells, the expression of a gene annotated as coding for Pumilio homologue 5 increased approximately 2.5-fold under heat shock conditions (36 °C for 4 h) ([50], see supplementary data, Additional file 6). Su et al. [54] reported that one gene encoding a Pumilio domain-containing protein was significantly down-regulated in *C. goreaui* following one week of microplastic exposure. Additionally, in *K. brevis*, transcripts encoding Pumilio 1 homologue, along with other regulators of gene expression, showed a significant decrease in abundance throughout the stationary phase of culture growth [52]. Here it should also be noted that Roy and Morse [53] examined potential substrates for casein kinase 2, a kinase that can be implicated in circadian regulation, in *L. polyedra*. Among the proteins bearing confirmed phosphosites, they identified not only PUM-HD-carrying proteins but also the previously discussed RRM-, KH-, and ZF-domain-bearing RBPs, as well as the OB-fold family proteins described below. Thus, RBPs emerged as one of the major groups of potential phosphorylation targets for this kinase [53].

## 8. OB-Fold Proteins

The oligosaccharide-/oligonucleotide-binding (OB) fold group comprises proteins with structurally similar core framework—a Greek key β-barrel composed of five anti-parallel β-strands, i.e., an OB-fold domain [172,173] (Figure 1f). These proteins are found in all three domains of life, and owing to the plasticity of the loops connecting β-strands, individual OB-fold domains range in length from 70 to 150 amino acids. This structural variability allows them to bind diverse ligands, including nucleic acids, oligosaccharides, phospholipids, and proteins. Ligand binding typically occurs on a side surface of the β-barrel, with strands β2 and β3 forming the binding site (reviewed in [174,175,176]).

The overall level of sequence similarity between different OB-fold subgroups is low. Nevertheless, their ubiquity, the conservation of the ligand-binding face, and the same mode of recognition in nucleic acid-binding OB-fold proteins, coupled with the ancestral architecture reconstruction results, suggest that this and several other groups of β-barrel proteins share the same evolutionary origin, with diversification predating the last universal common ancestor (LUCA) [172,175,177,178,179,180].

Among OB-fold proteins, nucleic acid-binding proteins comprise the largest and most diverse subgroup [181,182]. Among the most extensively studied members of this subgroup known to bind RNA are the S1 domain-containing and cold shock domain-containing proteins.

### 8.1. S1 Domain-Containing Proteins

The S1 domain was originally described in the *Escherichia coli* ribosomal protein S1 [183,184] that binds both rRNA and mRNA (reviewed in [82,175]). Often a single protein bears multiple tandemly repeated S1 domains: results of the taxonomic distribution analysis indicate that while the number of domains in bacterial proteins typically does not exceed six, eukaryotic proteins can contain up to fifteen copies [185]. With that, the level of sequence conservation between S1 domains of a single protein can vary [186]. In bacteria, S1 domains are found in ribonucleases (including degradosome components), transcription factors, and proteins involved in adaptation to various stressors (reviewed in [187]). In eukaryotes they have been described in some of the exosome proteins facilitating RNA binding and degradation, in subunits of RNA polymerase II, as well as in proteins involved in ribosome biogenesis, and in an α-subunit of the eukaryotic initiation factor 2 (eIF2), essential for the translation initiation control (reviewed in [187]).

In the *E. coli* S1 protein, an RNA-binding site includes F19, F22, H34, D64, and R68 residues, located on the same side surface as in other typical OB-folds. The aromatic residues provide side chains for the interaction with the RNA nucleobases [184]. While these five residues are mostly conserved in bacteria, in archaeal and eukaryotic S1 domains only the first F residue and residues D and R remain conserved [187]. These observations highlight a significant level of divergence characteristic to S1 domains.

Despite specific studies of the S1 domain-containing proteins of dinoflagellates are virtually absent, S1 domain-containing proteins and/or individual S1 domains were predicted and/or identified in both the genomic/transcriptomic and proteomic databases of different dinoflagellate species (e.g., [13,26,43,44,56]). In the *L. polyedra* nuclear proteome, RBPs containing from one to four S1 domains were identified; also, at least two proteins contained S1 together with other functional domains [28].

### 8.2. Cold Shock Domain-Containing Proteins

The cold shock domain (CSD), found in eukaryotic proteins, is homologous to bacterial cold shock proteins (reviewed in [188]), originally described in *E. coli* [189]. Whereas bacterial cold shock proteins typically consist of a single cold shock domain, CSD-containing proteins (CSD proteins) commonly possess multiple CSDs or additional functional modules such as basic/aromatic (BA) islands and zinc-finger domains [188]. Despite differences in structural organization and distribution across the domains of life, both bacterial cold shock proteins and eukaryotic CSD-containing proteins are often referred to as CSPs (e.g., [59,190]). However, to avoid confusion, we use the acronym CSP strictly to denote bacterial cold shock proteins unless it is part of a specific protein name.

In bacteria, CSPs are expressed in response to cold shock and can act as transcription activators of cold-shock induced genes. They also enhance RNA stability and facilitate translation initiation by preventing the formation of RNA secondary structures and stabilizing ribosome binding (reviewed in [191]). In plants, CSD-containing proteins have likewise been shown to participate in cold stress response and acquisition of freezing tolerance, as well as in regulating seed germination and growth [190,192,193].

Within the CSD, strands β2 and β3 bear the canonical nucleic acid-binding motifs RNP1 [KR]G[FY][GA]F[VI]X[FY] and RNP2 [LI][FY][VI][GK][NG]L—or modified variants [KS]G[FKY]G[FL]IXX and [LIV][FQ][VAL]HX[STR], respectively (reviewed in [191]) (Figure 1f). Single stranded DNA or RNA binding occurs in a groove on the β-barrel surface, primarily through interactions between aromatic amino acid side chains and nucleobases. The nucleic acid backbone is not involved in the interaction, which may explain why CSDs display limited sequence specificity and can bind both ssDNA and RNA (reviewed in [191,194]). Interestingly, at both the level of RNP motif composition and overall domain topology, CSDs show some resemblance to the RRM domains. However, this similarity is generally regarded as a case of convergence rather than homology [195,196].

Dinoflagellates appear to have an extensive repertoire of CSD-containing proteins. In *L. polyedra*, they constitute a significant fraction of nucleic acid-binding proteins in the nuclear proteome [28]. In the transcriptome of *L. polyedra*, 68% of all DNA-binding domains (in this and several other studies CSDs are classified within this category) corresponded to CSDs [58]. In transcriptomes of two *Symbiodinium* species, CSD was listed as the most common domain (>60%) of transcription factors [57]; the same was noted for the *S. microadriaticum* transcriptome [50]. *P. glacialis*, another member of the order Suessiales, also showed a strong over-representation of CSDs (PF00313) in transcripts [33]. In *P. cordatum*, Kalvelage et al. [66] reported 59 genes encoding putative CSD-containing proteins. Consistently, in the supplementary data of the *P. cordatum* genome annotation published by Dougan et al. [26], 57 protein-coding sequences were annotated as “CSP” or “CSP-like” proteins. Moreover, genes encoding CSD-containing proteins in dinoflagellates frequently occur in tandem arrays (see Supplementary Data in [26,32,33]).

All reported dinoflagellate CSDs carry two specific RNP motifs, with consensus sequences KGFGFI (RNP1) and VFVHXX (RNP2), as inferred from published alignments [59,61,62,63,64]. Predicted tertiary structures of *P. cordatum* (syn. *P. minimum*) PmCSP1 and PmCSP2 indicate that dinoflagellate CSD-containing proteins also fold into the canonical Greek key β-barrel [63] (Figure 1f). Similar to bacterial CSPs, which can bind DNA as transcription factors in addition to their RNA-binding function [191], dinoflagellate CSD-containing proteins also exhibit the ability to bind both DNA (dsDNA and ssDNA) and RNA non-specifically, as demonstrated in *L. polyedra* and *F. kawagutii* [59,64]. Notably, they display a preference for ssDNA and RNA over dsDNA binding [59,64]; moreover, RNA appears to compete efficiently with DNA [64].

In dinoflagellates, Beauchemin et al. [59] distinguished several types of CSD-containing proteins based on domain architecture: (1) proteins containing a single CSD, (2) CSD plus a C-terminal glycine-rich (GR) domain, (3) CSD plus a C-terminal GR domain and a zinc-finger domain, and (4) proteins with multiple CSDs and one/several RRMs. Most sequences fall into the first two types [59], and CSD proteins predicted in various dinoflagellate species correspond to these categories [61,62,63] (Table 3).

To date, CSD-containing proteins have been annotated or identified in various dinoflagellate species with different life histories, although functional data on their activity in dinoflagellate cells remains scarce. The only direct evidence for the involvement of CSD proteins in the cold shock response comes from *P. cordatum*, where two genes encoding proteins with a CSD + GR architecture showed enhanced expression after exposure to low temperatures (shift down from 20 °C to 16 °C, 12 °C, or 4 °C for 3 h or 6 h) [63]. Stephens et al. [45] listed CSD (PF00313) among the over-represented domains in the transcriptomes of psychrophilic dinoflagellates compared with transcriptome datasets from all other studied species. However, no significant changes in CSD-containing proteins expression were observed during cold-induced temporary cysts formation in *P. cordatum* [36] or *L. polyedra* [59,60]. In *S. acuminata*, a gene encoding a CSD protein did not show altered activity in response to low-temperature treatments [61], but such genes were significantly up-regulated in resting cysts [34,61]. In the early-branching dinoflagellate *Amoebophrya* sp., several genes coding for CSD proteins were most active at the free-swimming infective dinospore stage and during the maturation stage inside the host cell [43]. In *Alexandrium pacificum*, the expression of a gene encoding a protein with a CSD + GR + ZF domain composition increased both during the logarithmic growth phase and induced logarithmic growth phase (under high-phosphate/high-manganese conditions) [62].

## 9. DEAD/DEAH-Box Helicases

The DEAD/DEAH-box helicase family belongs to the superfamily 2 (SF2) of ATP-dependent, nucleic acid-binding RecA-like helicases, together with related groups such as DEAH-, DEXH-, and DEXD-box proteins [197]. This long-established and large protein family is widely distributed across all eukaryotes, most prokaryotes, and also in viruses [198,199,200].

A hallmark of DEAD-box helicases is the complex organization of the helicase core, which is its key functional region. The auxiliary N- and C-terminal extensions are generally non-conserved. The canonical helicase core contains nine conserved motifs responsible for ATP binding and hydrolysis and for RNA binding: the Q motif and motifs I, Ia, Ib, and II-VI [201] (Figure 1g). Additionally, conserved coupling elements referred to as Ic, IVa and Va are distinguished, giving a total of 12 conserved motifs [200,202]. Motifs I and II—Walker motifs A and B, respectively—are responsible for binding and hydrolysis of ATP. Motif I (AXTG[ST]GKT) forms the phosphate-binding loop, while motif II (DEAD), which gave the family its name, hydrolyzes bound ATP; these two motifs act in coordination with the Mg^2+^ ion [201]. The Q motif, specific to DEAD-box helicases (and absent in DEAH- and DEXH-box helicases), recognizes the adenine base of ATP. It interacts with the ligand-bound motif I and is considered to regulate ATPase activity by ensuring proper substrate binding [197,201]. Motif VI has also been proposed to bind the phosphoryl group of ATP and interact with motif II. Motifs involved in RNA binding include motif V (TDV[AG]ARGID), assisted by motifs Ia–Ic, and motif IV (minimal sequence reported [LV]IF) with IVa [200,201]. RNA binding sites contact the sugar phosphate backbone of RNA. Motifs III (SAT) and Va (when defined, extends into part of motif V and several downstream residues) are considered as linking ATPase and helicase activities. Structurally, the helicase core adopts a two-domain architecture (domains 1 and 2): each domain is composed of five β-strands and five α-helices (Figure 1g). These domains are connected by a flexible linker and form a cleft that constructs the ATP-binding site. The RNA binding site is located on the opposite site, perpendicular to the cleft.

The helicase core provides the ATP binding/hydrolysis cycles that are accompanied with conformational changes and RNA binding followed by unwinding. In addition, RNA clamping and annealing activities were shown for some helicases, as well as the ability to remove other proteins bound to RNA [200,201,202]. Functionally, DEAD-box helicases participate in a wide range of cellular processes associated with RNA metabolism [202]. A well-known member of this family is the eukaryotic initiation factor 4A (eIF4A), a subunit of the eIF4F complex that promotes translation initiation in eukaryotes [203]. The eIF4A helicase is required for exposure of mRNA to the ribosome. Besides, various DEAD-box helicases are involved in pre-mRNA splicing, ribosome biogenesis, RNA export, and degradation, as well in the processes providing organelle gene expression [199].

Dinoflagellates, like other eukaryotes, are expected to possess a variety of DEAD-box helicases. The motifs within the helicase core described above are highly conserved and well defined in their sequences, consistent with those in other organisms (Figure 1g). A rapid search for dinoflagellate DEAD-box including hits in the UniProtKB database (filters: “taxonomy_id:2864” AND “ft_domain:DEAD”) confirms this conservation. Literature analysis shows that DEAD-box helicases have been annotated or identified in genomic/transcriptomic/proteomic data from both basal and core dinoflagellate groups [26,43,44,45,46,50,52,66]. Besides, some genes coding for DEAD-box helicases are listed among those organized into tandem arrays (see Supplementary Data in [26,32,33]).

Proteomic analysis revealed a high abundance of DEAD-box RNA-helicases, including homologue of the splicing-dependent multiprotein exon junction complex component eukaryotic initiation factor 4A-III, among other nuclear proteins in the nuclear fraction of *P. cordatum* cells [66]. Similarly, Beauchemin and Morse [28] reported a large number of DEAD-box helicases among other RNA-binding proteins in the nuclear proteome of *L. polyedra*. They also presented schematic representations of the revealed RBP domain architecture variants, although without detailed discussion. The predominant compositions included a single DEAD-box domain or a combination of DEAD-box and helicase C, the latter sometimes distinguished as encompassing motifs V and VI. Besides, proteins with DEAD-box + helicase C + HA2 + OB structure were identified [28]. The HA2 appear to be a helicase-associated domain, while the OB represents the aforementioned OB-fold domain; such an architecture resembles that of DEAH-box helicases, for example, the yeast RNA helicase-like Prp2 (precursor RNA processing) factor involved in pre-mRNA splicing [204]. Another identified composition, DEAD-box + helicase C + rRNA + HCT [28], may be interpreted as a DEXD/DEXH-type helicase containing rRNA-processing arch domain essential for proper 5.8S rRNA processing and a helicase C-terminal tail domain. Such a protein may be homologous to the RNA exosome complex cofactor Dob1/Mtr4 helicase [205].

Special mention should be made of proteins reported to possess the “RRM + DEAD” architecture [28]. We could not find any data on such proteins in dinoflagellates; moreover, this configuration appears to be rare among eukaryotes. In the GenBank and RefSeq NCBI databases, we found several DEAD-box sequences with predicted RRM/RRMs from some plants and tardigrades (for example, *Brassica napus* CAF1933040.1 and *Ramazzottius varieornatus* GAU95793.1), as well as a DECD-box chlorophyte sequence (*Bathycoccus prasinos* XP_007510271.1). However, no information about such helicases could be found in the literature. In prokaryotes, DEAD-box helicases with a single C-terminal RRM domain—such as *E. coli* DbpA and *Bacillus subtilis* ortholog YxiN, both involved in large ribosomal subunit biogenesis—have been described [206,207,208]. These proteins bind 23S rRNA by means of their RRM domain with high affinity and specificity; this interaction defines conformational changes that promote formation of the catalytically important, closed conformation of the protein. In turn, the DEAD-box helicase core acts as the catalytic domain that destabilizes RNA duplexes in an ATP-dependent manner [209,210].

**Figure 2:**
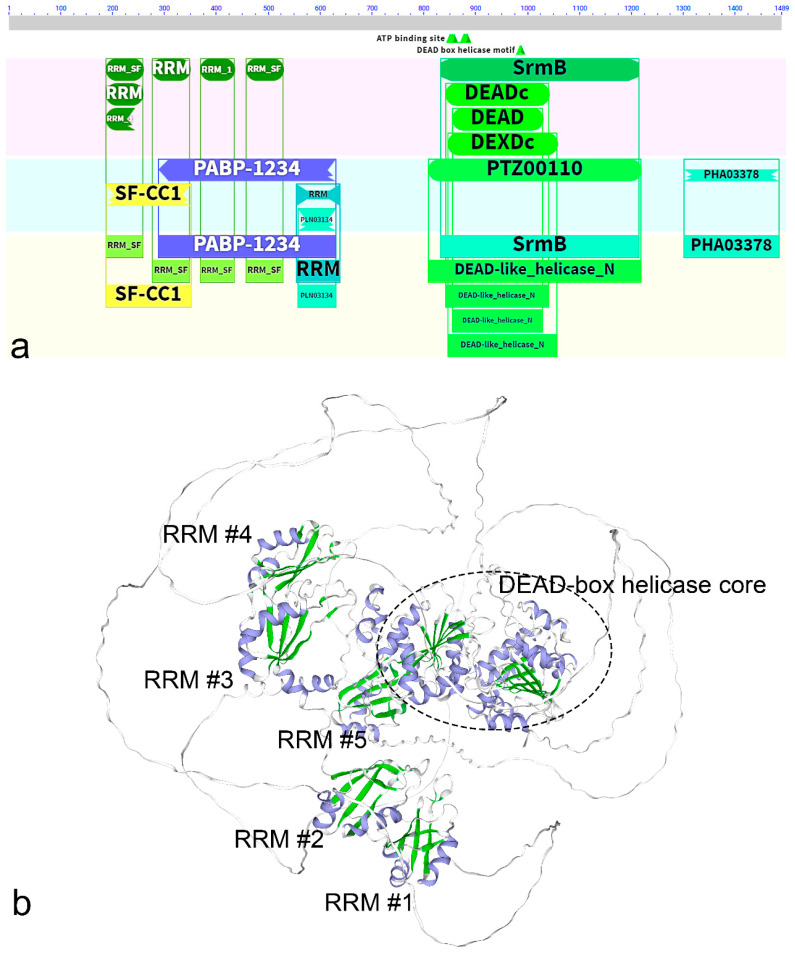
A putative RRM-DEAD protein (*Symbiodinium* sp. CCMP2456 sequence CAE7718496.1 retrieved from the GenBank database): (**a**) domain composition determined the using NCBI CD-search tool and (**b**) tertiary structure predicted by the AlphaFold Server. In (**b**), the colour scheme reflects secondary structure features: α-helices (lilac) and β-sheets (green). Five putative RRM domains and a DEAD-box helicase core (indicated by a dashed oval) are recognizable. Notably, the CD-search tool (**a**) determined four RRM domains as specific hits (upper pink-shaded panel), i.e., top-ranking hits that meets or exceeds a domain-specific E-value threshold, and a fifth RRM domain as a non-specific hit (middle blue-shaded panel), i.e., hits that meet or exceed the blastp threshold for statistical significance.

We searched for dinoflagellate proteins characterized by the presence of RRM and DEAD/DEAH-box helicase domains in the UniProtKB database, and then performed blastp analysis in the NCBI databases and MMETSP project transcriptome assemblies [211]. We revealed genomic and/or transcriptomic sequences—one sequence per assembly, with one exception—belonging to representatives of the Symbiodiniaceae and Kareniaceae families. These sequences possess a well-defined DEAD-box helicase core (with the DEAD signature) and upstream (rather, N-terminal in some cases) RRMs (e.g., *S. microadriaticum* OLP92746.1, *S. necroappetens* CAE7876399.1, *Symbiodinium* sp. CCMP2456 CAE7718496.1, *K. brevis* CCMP2229 20130916|87251_1) (Figure 2a). The number of RRMs varied depending on the prediction tool used and among taxa. In most symbiodiniacean sequences, the CD-search tool recognized four RRMs as specific hits (Figure 2a), although variants with one to three RRMs were also found. The InterPro [212] predictive models appeared to be more sensitive—or more permissive—identifying five RRMs in almost all sequences. Notably, the tertiary structure model predicted by the AlphaFold Server for *Symbiodinium* sp. CCMP2456 CAE7718496.1 did reveal five RRM-like βαββαβ folds (Figure 2b). In kareniacean proteins, four RRM domains were predicted by InterPro (and two to three by CD-search). The regions corresponding to the conserved RRM motifs RNP1 and RNP2 were modified relative to the canonical amino acid composition in the revealed sequences. This variation was especially pronounced in RNP2, while RNP1was conserved in some domains—e.g., KGFAYVQY motif—or retained a similar pattern. Again, as exemplified by the case above, the characteristic βαββαβ fold of RRM domains was preserved.

Additionally, we revealed similar sequences in transcriptome assemblies from *S. acuminata* and the heterotrophic dinoflagellates *Tripos fusus* (syn. *Ceratium fusus*) and *C. cohnii*. In these proteins, only one RRM domain was recognized. Moreover, the *S. acuminata* and *T. fusus* sequences contained one N-terminal ZF domain of the CCHC type with a motif CX_2_CX_3_HX_4_C (Figure 1d), which is slightly different from the typical CX_2_CX_4_HX_4_C motif, characteristic of the CCHC ZF domains [157,162]. Among DEAD-box helicases, homologues of Vasa identified in invertebrates (except for insects) are known to contain CCHC-type ZFs [213]. Vasa proteins were primarily characterized as functioning during the germ line development and participating in the germ cells specification, proliferation, and maintenance via translation regulation. Besides, in various taxa, genes encoding Vasa homologues are expressed in non-germ cell types such as multipotent stem cells [213]. The ZF domains are believed to contribute to RNA target specificity and may be linked to Vasa functions beyond the germ line [213]. Interestingly, among Vasa homologues we also found DEAD-box helicases with RRM domains. ZF-containing Vasa-2 homologues from hydrozoan cnidarians, such as *Hydra vulgaris* (BAB13308.1), *Nanomia bijuga* (AHI50305.1) [213,214] exhibited N-terminal RRMs, although these domains were not described in the related studies. Their Vasa-1 homologues by contrast, lack RRMs and are phylogenetically distant from Vasa-2 [214].

The conserved composition of the helicase core suggests that DEAD-box helicases in dinoflagellates retain their functional activity. As for their role in cellular processes, available data points, at minimum, to correlations of their activity with cell cycle progression and growth regulation. In *K. brevis*, genes encoding DEAD-box helicases were repressed during the stationary phase of culture growth [52]. Besides, a DEAD box RNA helicase was mentioned as belonging to a cluster of genes, which were up-regulated throughout the light phase of the diel cycle and characterized by a moderate amplitude of expression change during this phase [65]. In *L. polyedra*, two DEXD- and DEXH-containing proteins were overrepresented in the light phase (midday) samples [28]. A DEAD/DEAH box helicase domain-containing protein was highly expressed during the G2/M and, presumably, S phases of the cell cycle in *Prorocentrum donghaiense* [56].

Notably, information on the dinoflagellate homologues of the eukaryotic initiation factor 4A (eIF4A), unlike other translation initiation factors, is very scarce. Mentions of its presence appear in omics datasets [46], and several eIF4A annotated entries exist in the UniProtKB database.

## 10. YTH Domain-Containing Protein Family

Members of the YT521-B homology (YTH) domain-containing protein family share a conserved domain with the mammalian YT521-B splicing factor and are now known to be broadly distributed across eukaryotes [215,216]. Unlike many other RBPs that occur in both prokaryotes and eukaryotes, YTH domain-containing proteins are exclusive to eukaryotic organisms [216].

Most YTH proteins possess a single C-terminal YTH domain of approximately 145–150 amino acids, typically accompanied by intrinsically disordered N- or C-terminal regions [215,217]. The YTH domain adopts a mixed α-helix/β-sheet fold (Figure 1h), organized into a four α-helices and six β-strands [215] or three α-helices, eight β-strands, and two 3_10_-helices [218]. A defining structural feature of YTH domain is an aromatic cage that is composed of conserved Trp and Phe residues located within β-strands; this structure is essential for RNA binding [216,217]. The YTH domain binds single-stranded RNA [216]. The YTH domain-containing proteins have been characterized as so-called m^6^A readers—proteins that specifically recognize N^6^-methyladenosine (modified adenosine in RNA). The methyl group is accommodated within the aromatic cage and additionally stabilized by residues forming hydrogen bonds with the nucleotide [219,220].

YTH domain-containing proteins are key effectors of the epitranscriptomic m^6^A pathway and participate in multiple aspects of RNA metabolism. They regulate mRNA stability and degradation, splicing, translation, and nuclear export, thereby facilitating rapid remodeling of gene expression programs (see for review [217]). Notably, in fission yeasts, YTH domain-containing protein Mmi1 binds transcripts of meiosis-associated genes to prevent entry into the meiotic program; this repression is later relieved through inactivation of Mmi1 by the RRM-containing protein MEI2 and the long non-coding meiRNA during meiotic initiation [90,221,222].

In dinoflagellates, in contrast to other RBPs, only fragmentary data are available regarding the presence of YTH domain-containing proteins. Two genes encoding proteins with YTH have been annotated in the *P. cordatum* genome (Supplementary Information in [26]), and nine in the *A. minutum* genome [40]. These proteins—for example, *P. cordatum* CAK0851154.1—indeed contain a well-defined C-terminal YTH domain with conserved Trp and Phe residues and exhibit a mixed α-helix/β-sheet fold similar to canonical structures (Figure 1h). Mentions of YTH domains in dinoflagellates are also found in Roy et al. [13] and Stephens et al. (Supplementary Information in [45]). Additionally, homologs of the YTH-containing DEXH-box RNA helicase YTHDC2 have been predicted in the genomes of *Breviolum minutum* and *F. kawagutii* [44]. In mammalian cells, YTHDC2, similarly to yeast Mmi1, contributes to the transition from mitotic to meiotic divisions [223]. Notably, despite an extensive repertoire of MEI-like proteins, dinoflagellate appear to lack any recognizable homologs of Mmi1 protein.

The activity and cellular functions of YTH-containing proteins in dinoflagellates remain entirely unexplored experimentally. In this context, it is particularly intriguing that in these algae, m^1^A mRNA modification, rather than m^6^A, has been reported as the most prevalent internal mRNA modification [224]. This raises the question of whether dinoflagellate YTH proteins interact with RNA in the same manner as their counterparts in other eukaryotes, or whether their ligand specificity and functional roles may differ substantially.

## 11. Pentatricopeptide Repeat Proteins

The pentatricopeptide repeat (PPR) proteins form a large family of sequence-specific RNA-binding factors, which is widely expanded among eukaryotes. They are structurally similar to tetratricopeptide repeat (TPR) proteins, which are engaged in protein–protein interactions and occur in both prokaryotes and eukaryotes [225]. PPRs are thought to have evolved from TPRs early in eukaryotic history (see for review [226]).

The structural basis of PPR proteins is the PPR motif, a ~35-amino acid (canonical type) repeat arranged in tandem. The repeat is degenerate, i.e., showing diverse amino acid combinations while retaining key structural and functional characteristics [226,227]. The motif conserved patterns include coordination of nucleotides by charged or polar residues and the presence of a hydrophobic pocket containing a proline residue between two adjacent motifs [72,228]. To describe RNA recognition specificity, the concept of a “PPR code” was introduced—specific combinations of amino acids at two key positions within the PPR motif that determine binding to a particular nucleotide [226]. According to the current plant “code”, the fifth and last residues of the 35-amino-acid repeat are primarily responsible for nucleotide recognition [72]. The most common combinations at these positions include Asn and Asp, though Thr and Ser also occur frequently [72].

Each PPR motif adopts a helix-turn-helix hairpin structure consisting of two antiparallel α-helices, helix a and helix b [72,228,229]. Tandem motifs stack into a right-handed α-solenoid, forming a central groove that binds RNA [72,228] (Figure 1i). The RNA-binding fifth residue corresponds to the helix a, while the last residue lies in the loop interconnecting adjacent motifs [72]. PPRs bind single-stranded RNA in a one-repeat: one-nucleotide manner. Experimental and computational analyses have defined a correspondence between residue pairs and target nucleotides (e.g., [230]). Compared to other RNA-binding proteins, PPRs recognize target sequences of a greater length and diversity (see for review [226]).

Based on their repeat architecture, the PPR family is conventionally divided into two major subfamilies (classes). P-class proteins consist solely of canonical 35-residue P motifs and may lack additional domains; however, several subgroups can be distinguished in which the PPR array is followed by C-terminal domains, such as PPR-SMRs carrying small MutS-related (SMR) domain, PRORP-PPRs with a metallonuclease domain, PPR-TGMs with tRNA guanine-N7 methyltransferase domain, or PPR-TGMs that also possess CCCH-type ZF [231].

The PLS-class PPRs contain a characteristic triplet composed of P-, L- (long; ~35–36 amino acids) and S- (short; ~31–32 amino acids) repeats. They are arranged as (P1–L1–S1)^n^ followed by a P2–L2–S2 triplet and, in some cases, can alternate with or be preceded by SS-type repeats. All PLS-associated repeat variants differ in length and in some positions; for example, P1 and P2 repeats differ from the canonical P repeat in the amino acid composition of the first helix [72]. Subgroups of PLS proteins may also harbor downstream E1/E2-repeats [72]. DYW-type PPRs carry an extended C-terminal DYW region, which includes a zinc-dependent cytidine deaminase signature implicated in cytidine-to-uridine RNA editing [232,233].

Although encoded in the nucleus, most PPR proteins are targeted to mitochondria or plastids, where they act as key post-transcriptional regulators of organellar gene expression by participating in RNA stabilization, splicing, processing, translation, and editing [226,231,234,235,236].

PPR proteins appear to be well-defined (see as an example Figure 1i) and are reported to be highly abundant in dinoflagellates. Moreover, genomic evidence indicates they are definitely among the PPR-coding genes arranged into blocks of tandemly duplicated genes (see Supplementary Data in [26,32,33]). The abundancy of PPRs has been shown for species from various taxa: PPR domains were among the most numerous in the genomes of *Amphidinium gibbosum* [75], as well in symbiodiniacean and suessiacean species [11,33,44,45,51,70]. In thecate photosynthetic species, several hundred putative P-class PPRs can be distinguished: 676 and 536 genomic-based protein sequences were reported for *P. glacialis* and *P. cordatum*, respectively [78]; more than 200 transcriptome shotgun assembly entries were reported for *Alexandrium tamarense*, and about 800 for *L. polyedra* [69] (Table 4). Symbiodiniaceans appear to demonstrate comparable numbers of PPRs: 556 and 265 PPR-containing gene models were annotated for *S. microadriaticum* and *F. kawagutii*, respectively [44] (Table 4). For *B. minutum*, the number of annotated P-class PPR-coding genes varies from 493 [44] to 620 [23]. Cheng et al. [72] listed 687 (per the main text)/784 (per the supplementary data) P-class PPRs in *B. minutum* (Table 4).

Notably, the athecate dinoflagellate *K. brevis* (Gymnodiniales) possesses about 100 [67] or 120 [69] PPRs (Table 4). If confirmed by broader datasets, this may indicate that the major expansion of PPR-coding genes occurred after the divergence of Gymnodiniales. In addition, non-photosynthetic dinoflagellates—which are thought to retain a cryptic plastid [237,238,239]—also possess fewer but still substantial numbers of PPRs. Transcriptome data from *C. cohnii* revealed 57 P-class PPRs, and even the early-branching basal species *O. marina* has 46 homologues [78] (Table 4).

The diversity of dinoflagellate PPRs appear to be largely formed by P-class proteins, and, moreover, the main pool likely consists of “sensu stricto” P-class PPRs that lack additional domains. Nevertheless, dinoflagellate PPR repertoires may also include proteins belonging to other PPR subgroups. Core dinoflagellates possess PPR-SMRs [78]—P-class PPR proteins with a C-terminal SMR domain, presumably an endonuclease [240]. According to plant studies, PPR-SMRs can participate in organellar RNA stability, translation, or editing [240,241,242,243]. Dinoflagellate PPR-SMRs fold into the typical superhelical PPR structure, and their SMR domain exhibits the characteristic βαβαββ topology [78]. In general, two conserved motifs typical of SMR domains—the N-terminal X_a_DXH (with X for any nonpolar amino acid residue and X_a_ for only aliphatic ones) and the putative catalytic TGXG site—are retained, although some individual substitutions may occur [78]. Importantly, dinoflagellates have seized the SMR domain fused to the PPR backbone, whereas dinoflagellate homologues of MutS2—the other protein canonically bearing the SMR domain—exist as MutS domain III-V-containing MutS2-like proteins and lacking an SMR domain [78,244]. Across different species, usually 1–2, and in some cases 4–5, PPR-SMR homologues are present (Table 4). Phylogenetic analyses based on alignments of PPR-motif regions show that they do not separate from the broader diversity of dinoflagellate P-class PPRs without additional domains [78]. Notably, dinoflagellates with tertiary plastids have one PPR-SMR each: sequences from kryptoperidiniacean species with diatom-derived plastids cluster with diatom whereas those from kareniaceans with haptophyte-derived plastids branch together with dinoflagellate PPR-SMRs [78].

The presence of PPRs from other P-class subfamilies in dinoflagellates has yet to be proven. Nevertheless, for example, brief screening of the published *P. cordatum* genomic data already indicates the existence of PPRs with a putative methyltransferase domain [26]. PPR-TGMs with tRNA guanine-N7 methyltransferase domain have been predicted in various protists, including the alveolate *Perkinsus marinus* [235,245]. These proteins are predominantly targeted to mitochondria—plastid targeting remains unclear—and are predicted to function in tRNA metabolism [245].

It should also be noted that among P-class PPRs (both “sensu stricto” and PPR-SMRs), sequences containing a specific and previously undescribed Ser-Ala-Cys-Glu-Lys (SACEK) motif were reported in core dinoflagellates [78]. It occupies the pre-turn region of helix a of the 35-amino-acid PPR hairpin (positions 9–13). Outside dinoflagellates, such sequences were found only in heterokontophytes and in some cryptophyte and haptophyte species—i.e., among lineages of the CASH assemblage. Despite some occasional substitutions (most frequently at position 12), the SACEK motif is highly conserved; in addition, a downstream Trp and several nonpolar residues are generally well conserved. In some sequences, multiple consecutive SACEK-containing PPR repeats were observed. These SACEK-PPRs are abundant, for example, in *P. cordatum*, *P. glacialis*, and *Alexandrium tamarense*. In other taxa, only one to three SACEK repeats (or modified variants) were found. The species listed above also contain such homologues, whereas others (*A. carterae*, *S. acuminata*, *C. cohnii*) possess only these SACEK-PPRs. Phylogenetically, proteins with multiple SACEK repeats form a well-supported group; most SACEK-containing sequences, regardless of the composition, tend to cluster on closely related branches. It is noteworthy that dinoflagellate P-class PPRs lacking SACEK signature group separately; in one such cluster, they group together with other alveolates [78]. Given the location of the motif opposite to the putative nucleotide-recognition site and its amino acid properties, the SACEK motifs may contribute to stabilizing α-helical stacking within the PPR superhelix or modulating its flexibility [78].

The presence of PLS-class PPRs in dinoflagellates remains questionable. As noted above, Cheng et al. [72] reported 784 P-class PPRs in *B. minutum* within their analysis of putative PPR motif structures across 109 eukaryotic species. According to their results, many *B. minutum* PPRs contain not only canonical P repeats but also arrays of P1 or P2 repeats (611 gene-based variants of predicted motif structure in total) (Table 4). Although P1 and P2 repeats were originally described in the context of PLS-class PPRs, these variants are more appropriately classified as P-class PPRs. Besides, 173 genes encoding combinations that also include L1, L2, S1, S2, or SS repeats were listed (Table 4). However, the strict repetitive P-L-S triplets organization required for assigning proteins to the PLS-class was not observed in *B. minutum* sequences. Furthermore, E-type variants were absent [72].

Interestingly, in their study of RNA editing under different stress conditions in *S. microadriaticum*, Liew et al. [73] mentioned two candidate PPR proteins with presumable DYW domains as candidates for components of the RNA-editing machinery. Two PPR genes containing DYW domain were also mentioned in the *B. minutum* genome [23,72] (Table 4). On the one hand, the published alignment of the corresponding region of *B. minutum* sequences with the DYW domain from *Arabidopsis thaliana* proteins [73] shows that the examined dinoflagellate sequences contain the highly conserved cytidine deaminase signature HXEX_n_CXXC, a key Zn^2+^-binding motif required for RNA editing [246]. On the other hand, they lack the C-terminal Asp-Tyr-Trp (DYW), or homologous, signature essential for domain activity [73]; only the sequence symbB1.v1.2.035968.t2 contains residues Asn-Glu-Trp (NEW) at the corresponding position (Figure S3). Moreover, our analysis of the full corresponding *S. microadriaticum* and *B. minutum* sequences retrieved from GenBank, ReefGenomics (http://reefgenomics.org/, accessed on 29 October 2025), and the OIST Marine Genomics Unit (https://marinegenomics.oist.jp, accessed on 29 October 2025) databases revealed, in some cases, an extension downstream of the putative DYW domain containing ankyrin repeats (Figure S3). Only a few similar dinoflagellate sequences can be found in currently available datasets. Therefore, the presence and functional significance of putative DYW-type PPR proteins in dinoflagellates remains an open question.

**Figure 3:**
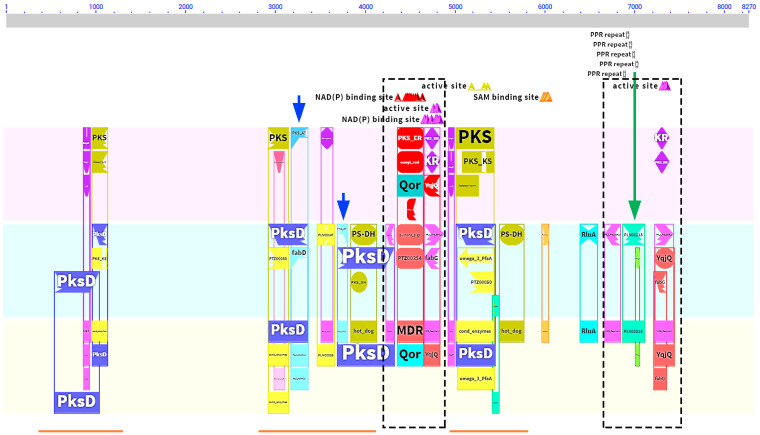
A putative *cis*-polyketide synthase protein containing a ketoreductase unit with several PPR repeats (*Symbiodinium microadriaticum* sequence OLP78851.1 retrieved from the GenBank database), domain composition determined using the NCBI CD-search tool. Putative modules containing PKS-associated domains are underlined with orange lines, blue arrows indicate acyl transferase domains, green arrows—PPR repeats, ketoreductase units are indicated by dashed boxes.

Another curious finding concerning PPR domains in dinoflagellates comes from the study of polyketide synthases (PKS) and non-ribosomal peptide synthases (NRPS) diversity in representatives of three symbiodiniacean clades—B1, A3, and C (*B. minutum*, *Symbiodinium tridacnidorum*, and *Cladocopium* sp., respectively) [74]. PKS and NRPS are modular enzymes responsible for the biosynthesis of secondary metabolites [247]. The authors noted that PPR repeats, together with ankyrin and HEAT repeats, may occur as accessory units fused with certain monofunctional PKS or NRPS domains, presumably *trans*-acting ones, i.e., encoded separately from the other domains in the module in the biosynthetic gene clusters [248]. We were not able to identify such PPR-containing sequences on the basis of the published materials (in contrast to HEAT- or ankyrin-containing ones). However, a search for PKS/NRPS sequences with PPR domains in public databases revealed two *S. microadriaticum* PKS proteins (OLP78851.1 and CAE7327639.1). As an example, Figure 3 shows the domain organization of the protein OLP78851.1. They consist of putative modules—three modules in OLP78851.1 and one in CAE7327639.1—containing PKS-associated domains in different combinations. Notably, OLP78851.1 possesses acyl transferase domains in these modules, suggesting that it represents a *cis*-PKS rather than a *trans*-PKS (Figure 3). At the C-terminus, both sequences contain an accessory region with a ketoreductase (KR) unit that includes several PPR motifs (Figure 3). These PPRs turned out to be SACEK-containing PPRs, with multiple consecutive SACEK-type repeats.

The KR module comprises two subdomains, a structural and a catalytical one, the latter containing the YXXXN active-site motif; an additional domain such as enoylreductase (ER) may be inserted between them [249]. Indeed, this arrangement is observed in the upstream module of the revealed *S. microadriaticum* PKSs. Interestingly, the KR with the inserted ER contains the canonical NADPH-binding motif TGGTGXLG, whereas the KR with inserted PPRs lack it and retains only the YXXXN motif, which may indicate it represents a different KR type [249]. We found no literature describing PPR repeats associated with a KR module. However, some plant PPR-containing proteins (mainly DYW-type PPRs) with a C-terminal domain belonging to the short-chain dehydrogenase/reductase (SDR) superfamily, to which KRs also belong [249], and with the YXXXK active-site motif, can be retrieved from databases.

PPR-containing PKSs seem to represent a rare enzyme architecture in dinoflagellates and in eukaryotes more broadly. A more common feature of PKSs in these protists is the presence of tetratricopeptide repeats (TPRs), which participate in protein–protein interactions [250]. TPRs co-occurring with thiolation (acyl carrier protein) domains have been described in core dinoflagellates [251,252]. Such combinations likely serve as scaffolds for protein domains and provide reaction centers for the large complexes that make it possible to synthesize the large toxins specific for core dinoflagellates [252]. In general, secondary-metabolite biosynthetic machinery is thought to evolve rapidly and extensively through mutations, module duplication, rearrangements, and related processes, resulting in remarkable diversity of the molecules produced [253,254,255]. The incorporation of PPR repeats into PKS aligns with this concept and may provide additional functional capabilities to the dinoflagellate biosynthetic apparatus. Nevertheless, the rarity and apparent uniqueness of this architecture suggest that such a fusion may represent an isolated event.

In *K. brevis*, activity PPR-coding genes appeared to be highly dynamic in response to changes in the nutritional conditions [67]. When in nitrate- or phosphate-depleted cultures, the nutrient levels have been restored, rapid upregulation of PPR genes was observed, preceding alterations in expression of genes related to the photosystem. Moreover, detected PPR transcripts have already undergone SL trans-splicing during this response [67]. Such PPR transcripts are characterized by short half-lives [68]. However, the level of de novo transcribed RNA coding for PPRs was low [68]. The expression of PPR-coding genes begins to decrease early in the stationary phase and continues to be repressed more and more throughout it [52]. In *B. minutum*, PPRs appeared to be suppressed in cells within the symbiosome of the coral host compared to cells cultivated as free-living algae [76].

Lin et al. defined PPR-coding genes as non-specific stress response genes [71]. The study of dinoflagellate cells that are exposed to various conditions evidence it. In the model of heat stress (one-week growth under 30 °C), five (gene IDs Skav218478, Skav218166, Skav226499, Skav228199, and Skav233181) out of seven differentially expressed PPR-coding genes were up-regulated and two genes (IDs Skav205856 and Skav204446) were down-regulated. In cells grown in the phosphate-depleted conditions or in medium with dissolved inorganic phosphate replaced by organic one, differential expression of these genes was also observed [71]. Notably, direction of changes was the same for the genes common for different stress conditions [71]. Cold stress also altered the expression of PPR-coding genes: 32 genes were significantly down-regulated in *P. cordatum* cold-induced temporary cysts, and, in turn, 29 ones became up-regulated during the preparation of cells for excystment (14 DEGs were common for these conditions) [36]. Exposure of *Cladocopium* sp. cells to nano-plastics in the cultivation medium reduced expression of genes coding for PPR domain-containing proteins [77].

## 12. Perspectives and Emerging Approaches in Dinoflagellate RBP Research

In general, the dinoflagellate RBPs discussed here demonstrate substantial conservation and high similarity to proteins considered canonical in other eukaryotes, suggesting that their core cellular functions have largely been retained; nevertheless, specific lineage-related features can be identified in several cases. Some RBP groups, such as PPR proteins and MEI2-like homologues, show extensive expansions in dinoflagellate genomes. In the case of PPR proteins, this expansion may be primarily related to the extensive RNA editing that occurs in dinoflagellate chloroplasts [23]. At the same time, the expansion of MEI2-like proteins and the suggested existence of putative functional groups may reflect diversification of post-transcriptional regulatory pathways associated with distinct life-cycle stages and adaptive responses to environmental stressors. Notably, genes encoding some RBPs (including PPRs, CSPs, and DEAD-box helicases) are often arranged in tandem arrays [26,32,33].

Despite the increasing amount of sequence-based information, mechanistic characterization of dinoflagellate RBPs remains limited. For most proteins, functional inference relies almost exclusively on homology-based annotation, with few biochemical or experimental studies addressing RNA targets, binding affinities, or physiological roles. Bridging this gap will require a shift from descriptive genomics toward targeted functional analyses.

Promising research directions encompass both relatively straightforward and more technically demanding approaches. These include detailed structural characterization of RBPs, determination of their intracellular localization, targeted analyses of RBP gene expression dynamics across environmental conditions and life-cycle stages, and comparative studies of free-living, symbiotic, and parasitic dinoflagellate lineages to identify evolutionary drivers underlying RBP family expansion.

Another largely unexplored direction concerns the potential involvement of dinoflagellate RBPs in liquid–liquid phase separation (LLPS) during transitions between life-cycle stages, particularly during resting cyst formation. LLPS is a major mechanism underlying the assembly of various membraneless cellular compartments, including nucleoli, Cajal bodies, stress granules (SGs), and processing (P) bodies (reviewed in [256]). The latter two compartments are composed predominantly of mRNA molecules and RBPs and are involved in mRNA sequestration and regulation of mRNA degradation. While SG formation is strictly stress-induced, P-bodies may be constitutively present in the cell [256].

Resting cyst formation in dinoflagellates is frequently triggered by environmental stressors such as nutrient limitation, changes in illumination, or temperature shifts; in many cases, encystment is also preceded by sexual fusion [4,6]. In this context, the increased expression of genes encoding CSD-containing proteins in dinoflagellate resting cysts [34,61] is particularly noteworthy, as it suggests that at least some of these proteins may participate in the intracellular processes that mediate rearrangements accompanying entry into dormancy. Notably, many proteins implicated in LLPS in other eukaryotes possess intrinsically disordered regions (IDRs) and/or multiple modular interaction domains [257], and some dinoflagellate CSD proteins either possess tandemly repeated domains or glycine-rich IDRs [59]. Together, these observations indicate that elucidating the roles of CSD-containing proteins and possibly other RBP groups during encystment, including their potential contribution to phase separation-mediated organization of RNA-protein assemblies, represents an important avenue for future research.

To understand how RBPs shape the unique biology of dinoflagellates and to uncover the broader principles of RNA metabolism across various eukaryotic lineages, further methodological advances are required. Emerging genetic transformation and functional genomics approaches are gradually making experimental investigation of gene function and regulatory mechanisms in dinoflagellates feasible. Recent studies have established a nuclear gene transformation protocol in the early-branching dinoflagellate *Oxyrrhis marina* [258], and in the coral symbiont *Breviolum minutum* [259], enabling stable transgene expression. In parallel, the dinoflagellate-specific trans-spliced leader, a defining feature of their mRNAs, is exploited to recover and analyze full-length transcripts, particularly from mixed microbial samples [260,261]. In addition, single-cell transcriptomic analysis of early-branching, uncultured dinoflagellates of the genus *Abedinium* has been applied not only to resolve their phylogenetic relationships but also to shed light on the evolutionary history of characteristic cellular features, including proteins involved in chromatin organization [262]. Together with high-throughput proteomics and integrative multi-omics approaches [26,46,263], these methods provide a framework for moving from descriptive genomics toward an understanding of functional role of distinct proteins and protein groups in life-history transitions, stress adaptation, and other cellular processes in dinoflagellates.

## Figures and Tables

**Table 1:** Major RNA-binding protein groups analyzed in this review.

RBP Group	Canonical Domain Composition	Fold Architecture	General Functions in Eukaryotic Cells	Level of Characterization in Dinoflagellates	Relevant Studies in Dinoflagellates
RRM domain-containing proteins (MEI2 and MEI2-like)	Three RRM domains *	β_1_α_1_β_2_β_3_α_2_β_4_ (the third RRM domain has a β_1_α_1_β_2_β_3_α_2_β_4_α_3_β_5_β_6_ fold)	post-transcriptional regulation of developmental gene expression, incl. via binding and control of non-coding RNA and mRNA stability, mRNA sequestration	Presence in genomic/transcriptomic data, DGE **, sequence analysis	[6,31,32,34,35,36,37,38,39,40]
RRM domain-containing proteins (PABP)	RRM domains + C-terminal PABP-specific domain	β_1_α_1_β_2_β_3_α_2_β_4_	mRNA circularization, polyadenylation, nuclear export, degradation	Presence in genomic/transcriptomic data	[26]
Sm/Lsm proteins	Sm domain with motifs Sm1 and Sm2 + linker	N-terminal α-helix + 5 antiparallel β-strands	mRNA splicing, chaperone activity, protection from exonucleases, promotion of mRNA decay	Presence in genomic/transcriptomic data, immunocytochemical labeling	[13,22,26,33,41,42,43,44,45,46] Sm-binding site: [22,47,48,49]
KH domain-containing proteins	Number of KH-domains per protein varies	Type I: β_1_α_1_α_2_β_2_β′α′ Type II: β′α′β_1_α_1_α_2_β_2_	transcription, mRNA processing, translation	Presence in genomic/transcriptomic/proteomic data	[13,26,44,46,50,51]
ZF proteins	Number of ZF-domains per protein varies	C2H2: ββα CCCH: short α-helices + 3_10_-helices CCHC: 2 short β-strands + zinc knuckle + short α-helix	RNA folding, processing, turnover	Presence in genomic/transcriptomic/proteomic data	[13,26,28,43,44,50,51]
PUF proteins	Pumilio homology domain (eight tandem motifs)	3 α-helices in a triangular configuration	promotion or repression of translation, mRNA stability regulation	Presence in genomic/transcriptomic/proteomic data, DGE	[13,26,28,43,44,46,50,52,53,54,55]
OB-fold (S1 domain-containing proteins)	S1 domain	Greek key β-barrel (5 anti-parallel β-strands)	facilitation of RNA binding and degradation, ribosome biogenesis, control of translation initiation	Presence in genomic/transcriptomic/proteomic data	[13,26,28,43,44,56]
OB-fold (CSD-containing proteins)	Cold shock domain with RNP1 and RNP2 motifs		enhancing RNA stability, facilitating translation initiation	Presence in genomic/transcriptomic/proteomic data, DGE, sequence analysis, experimental data (binding tests)	[26,28,32,33,45,50,57,58,59,60,61,62,63,64]
DEAD/DEAH-box helicases	helicase core, 8 or 9 conserved motifs (Q—in DEAD-box only, I, Ia, Ib, II-VI)	domain 1 + domain 2, each of 5 β-strands + 5 α-helices	translation initiation, splicing, RNA export, degradation, ribosome biogenesis, organelle gene expression	Presence in genomic/transcriptomic/proteomic data, DGE	[26,28,32,33,43,44,45,46,50,52,56,65,66]
YTH domain-containing proteins	C-terminal YTH domain	mixed α-helix/β-sheet fold (4 α-helices + 6 β-strands or 3 α-helices, 8 β-strands, and 2 3_10_-helices)	regulation of mRNA stability and degradation, splicing, translation, RNA nuclear export	Presence in genomic/transcriptomic data	[13,26,40,44,45]
PPR proteins	Pentatricopeptide repeats (~35 a.a.)	right-handed α-solenoid of helix-turn-helix hairpin structures	organelle gene expression (RNA stabilization, splicing, processing, translation, editing)	Presence in genomic/transcriptomic data, DGE, sequence analysis	[11,23,26,32,33,36,44,45,51,52,67,68,69,70,71,72,73,74,75,76,77,78]

* Only the third RRM domain in identified MEI2 homologues in myzozoans, including dinoflagellates. ** DGE—differential gene expression data.

**Table 2:** Two putative functional groups of genes encoding MEI2-like proteins in dinoflagellates: genes demonstrating transcriptional responses linked to vegetative life-cycle stages and stress, and those potentially associated with the sexual process. Based on Table 1 from [31], with modifications. Changes in gene expression: ↑—increase, ↓—decrease.

Dinoflagellate Species *	Conditions	Life Cycle Stage	*MEI2*-Like DEGs Number/Expression Changes	Simultaneously Up-Regulated Meiosis-Associated Genes	References
Vegetative life-cycle stage- and stress-related response					
*Effrenium voratum*	Photobleaching (heat stress, 32 °C)	Coccoid cells	5/↓	-	[37]
*Fugacium kawagutii*	Phosphorous deficiency (1 week)	n/a	2/↓	-	[71]
*Prorocentrum cordatum*	Cold stress (2 °C)	Temporary cyst	5/↓; (2/↑ during excystation)	-	[36]
*Scrippsiella acuminata*	50–60-day cultures	Mature resting cysts	10 (4/↑; 6/↓)	*SPO11*, *DMC1*	[34]
	Dark + cold stress (8 °C)	Temporary (pellicle) cysts	10 (9/↑; 1/↓)	*SMC3*	[38]
Potential sexual life-cycle phase-related response					
*Alexandrium minutum*	Nitrogen deficiency (72 h)	n/a	n/a/↑	*TOP2*, 47 enriched GO terms related to meiosis	[40]
*Karenia mikimotoi*	Bloom, natural conditions	n/a	n/a/↑	*RAD54*, *MSH2*, *RAD21*, *SPO11*, *DMC1*, *MND1*, *SMC3*	[6]
*Noctiluca scintillans*	Bloom, natural conditions	n/a	6/↑	*SMC1*, *SMC3*, *RAD24*, *HOP2*, *MND1*, *REC8*, *DMC1*, *MSH5*, *EXO1*, *MRE11*, *MER3*/*HFM1*	[39]
*Prorocentrum shikokuense*	Bloom, natural conditions	n/a	n/a/↑ (some—↓?)	*DMC1*, *SPO11*, *MND1*, *MSH6*, *RAD21*, *RAD51*, *EXO1*, *MNS1*, *SMC3*, *SMC2*, *SMC1*	[6]

* Here and in the following tables, we used currently accepted species names according to the AlgaeBase and WoRMS databases.

**Table 3:** Variants of CSD-containing proteins domain architectures in dinoflagellates and their occurrence in different species.

Domain Architecture	Dinoflagellate Species	References
CSD	*Lingulaulax polyedra*	[59]
	*Pfiesteria piscicida*	[62]
CSD + GR	*Alexandrium tamarense*	[59]
	*Karenia brevis*	[63] *
	*Karlodinium veneficum*	[62]
	*Lingulaulax polyedra*	[59]
	*Prorocentrum cordatum*	[63]
	*Scrippsiella acuminata*	[61] **; [63]
	*Symbiodinium* sp.	[59]
CSD + GR + ZF	*Alexandrium pacificum*	[62]
	*Lingulaulax polyedra*	[59]
RRM + (CSD)^n^ + RRM	*Lingulaulax polyedra*	[59]

* Wang et al. [63] presented this CSD-containing protein (GenBank accession number FK848095.1) as possessing CSD + ZF + GR domain architecture (see Figure 3 in [63]); however, this sequence appears to lack any recognizable ZF and contain only GR region. ** Deng et al. [61] described this CSD-containing protein as possessing only CSD; however, Wang et al. [63] presented it as also exhibiting a C-terminal GR region (see Figure 3 in [63]) and provided amino acid sequence evidence supporting this domain organization.

**Table 4:** Diversity, classification, and abundance of PPR proteins in dinoflagellates based on available genomic and transcriptomic data. The table summarizes the presence of PPR proteins belonging to different subgroups of the P and PLS classes in dinoflagellates. For each taxon, the number of predicted PPR proteins or PPR-coding genes is indicated together with the data source of—genome (G) or transcriptome (T); proteome datasets derived from genome assemblies (e.g., in *P. cordatum* and *P. glacialis* cases) are also denoted as “G”.

PPR Class	PPR Subgroup	Dinoflagellate Species	Number	Data Source	References
P	P-class PPR (without additional domains)	*Alexandrium tamarense*	>200	T	[69]
		*Breviolum minutum*	493	G	[44]
			620	G	[23]
			611 *	G	[72]
		*Crypthecodinium cohnii*	57	T	[78]
		*Fugacium kawagutii*	265	G	[44]
		*Karenia brevis*	100	T	[67]
			120	T	[69]
		*Lingulaulax polyedra*	800	T	[69]
		*Oxyrrhis marina*	46	T	[78]
		*Polarella glacialis*	676	G	[78]
		*Prorocentrum cordatum*	536	G	[78]
		*Symbiodinium microadriaticum*	556	G	[44]
	PPR-SMR	*Alexandrium tamarense*	2	T	[78]
		*Cladocopium sp.*	1	T	
		*Crypthecodinium cohnii*	2	T	
		*Karenia brevis*	1	T	
		*Karlodinium veneficum*	1	T	
		*Lingulaulax polyedra*	5	T	
		*Prorocentrum cordatum*	2	G	
		*Polarella glacialis*	4	G	
		*Tripos fusus*	1	T	
		*Scrippsiella acuminata*	2	T	
PLS?	PPRs with L1, L2, S1, S2, or SS repeats (no P-L-S triplet)	*Breviolum minutum*	173 *	G	[72]
PLS	DYW-PPR	*Breviolum minutum*	2 **	G, T	[23,72]
		*Symbiodinium microadriaticum*			[73]

* Cheng et al. [72] reported 687 P-class PPR genes in *B. minutum* (per the main text). In Supplementary data Table S4 [72], 784 gene-based variants of predicted motif structure excluding DYW-type genes are listed. Among these, 611 variants contain neither L- nor S-repeat types and consist only of P repeats (including P1 and P2 repeats, which we group together here, although P1 and P2 repeats were originally described for PLS-class PPRs [72]). The remaining 173 variants contain L1, L2, S1, S2, or SS repeats in various combinations. Nevertheless, these variants cannot be strictly classified as PLS-class PPRs due to the absence of canonical P-L-S triplets. ** Only one sequence (symbB1.v1.2.035968.t2) in the relevant databases contains the amino acid residues Asn-Glu-Trp (NEW) at the position corresponding to the DYW motif.

## Data Availability

No new data were created or analyzed in this study. Data sharing is not applicable to this article.

## Supplementary Materials

The following supporting information can be downloaded at: https://www.mdpi.com/article/10.3390/ijms27010462/s1.
