# Supplementary material for: RNA-Binding Proteins in Dinoflagellates
Source: Int J Mol Sci. 2026 Jan 1;27(1):462. doi: 10.3390/ijms27010462 (PMC12787238; doi:10.3390/ijms27010462)
Supplement: Supplementary file 1 [file ijms-27-00462-s001.zip › Figure S1.pdf]

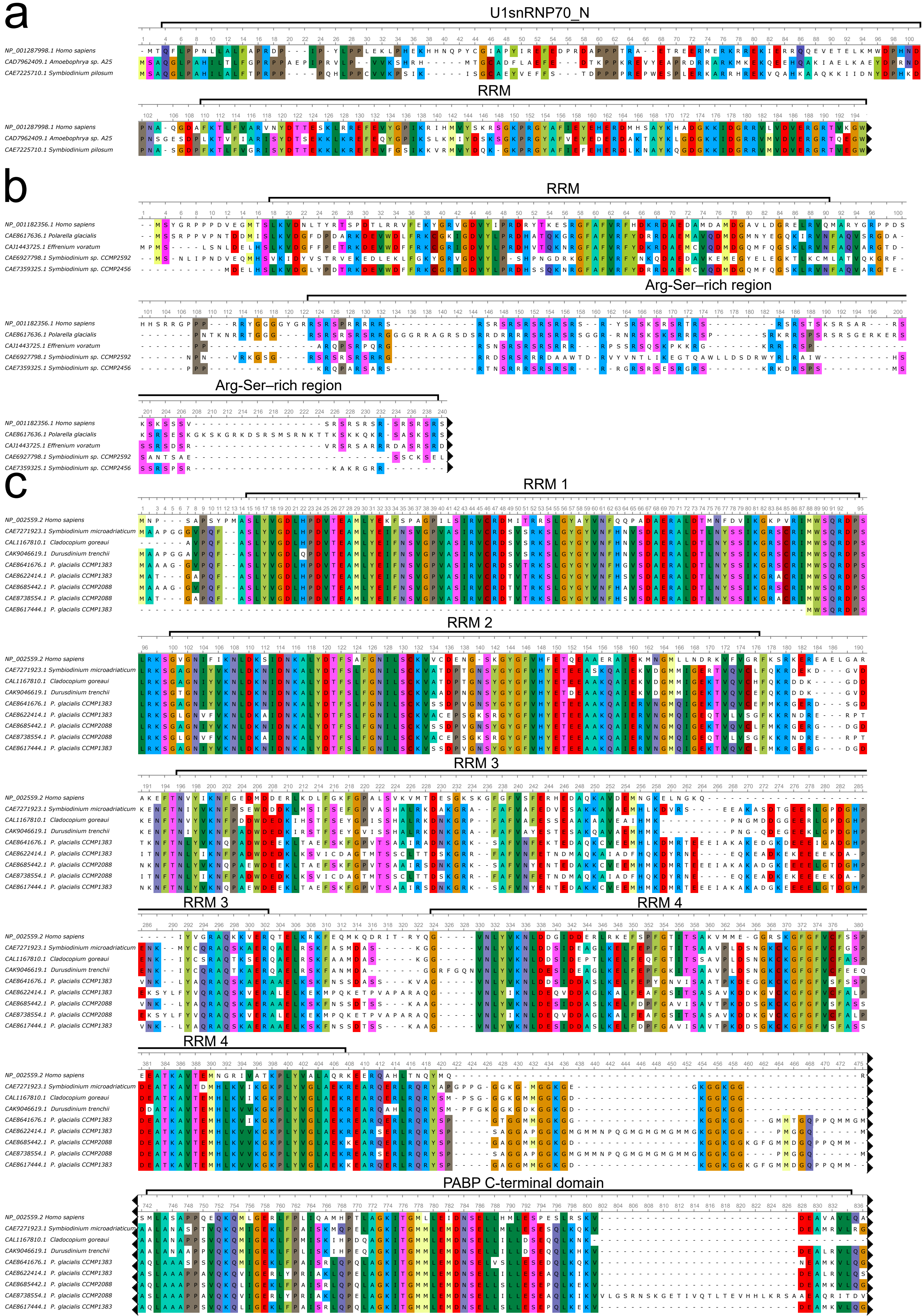
a
U1snRNP70_N
RRM
NP_001287998.1 Homo sapiens
CAD7962409.1 Amoebophrya sp. A25
CAE7225710.1 Symbiodinium pilosum
b
RRM
Arg-Ser–rich region
NP_001182356.1 Homo sapiens
CAE8617636.1 Polarella glacialis
CAJ1443725.1 Effrenium voratum
CAE6927798.1 Symbiodinium sp. CCMP2592
CAE7359325.1 Symbiodinium sp. CCMP2456
c
RRM 1
RRM 2
RRM 3
RRM 4
PABP C-terminal domain
NP_002559.2 Homo sapiens
CAE7271923.1 Symbiodinium microadriaticum
CAL1167810.1 Cladocopium goreaui
CAK9046619.1 Durusdinium trenchii
CAE8641676.1 P. glacialis CCMP1383
CAE8622414.1 P. glacialis CCMP1383
CAE8685442.1 P. glacialis CCMP2088
CAE8738554.1 P. glacialis CCMP2088
CAE8617444.1 P. glacialis CCMP1383

Figure S1. Multiple amino acid sequence alignments illustrating the domain composition of putative RRM domain-containing proteins of dinoflagellates, retrieved after the blastp searches. The alignments include non-exhaustive sets of (a) U1-70K homologues, (b) SRSF2 homologues, and (c) PABPC1 homologues. In each alignment, the first sequence corresponds to the human protein used as the query. To improve readability, the alignments were trimmed as follows: in (a), positions after 195 were removed; in (b), positions after 240 were removed; and in (c), region corresponding to positions 475–741 and positions after 836 were removed. The boundaries of the removed regions are indicated by a broken line. The boundaries of conserved domains are indicated above the corresponding regions of the alignments. Multiple sequence alignments were generated using MAFFT7 implemented in the Unipro UGENE software package.
