# Supplementary material for: RNA-Binding Proteins in Dinoflagellates
Source: Int J Mol Sci. 2026 Jan 1;27(1):462. doi: 10.3390/ijms27010462 (PMC12787238; doi:10.3390/ijms27010462)
Supplement: Supplementary file 1 [file ijms-27-00462-s001.zip › Figure S2.pdf]

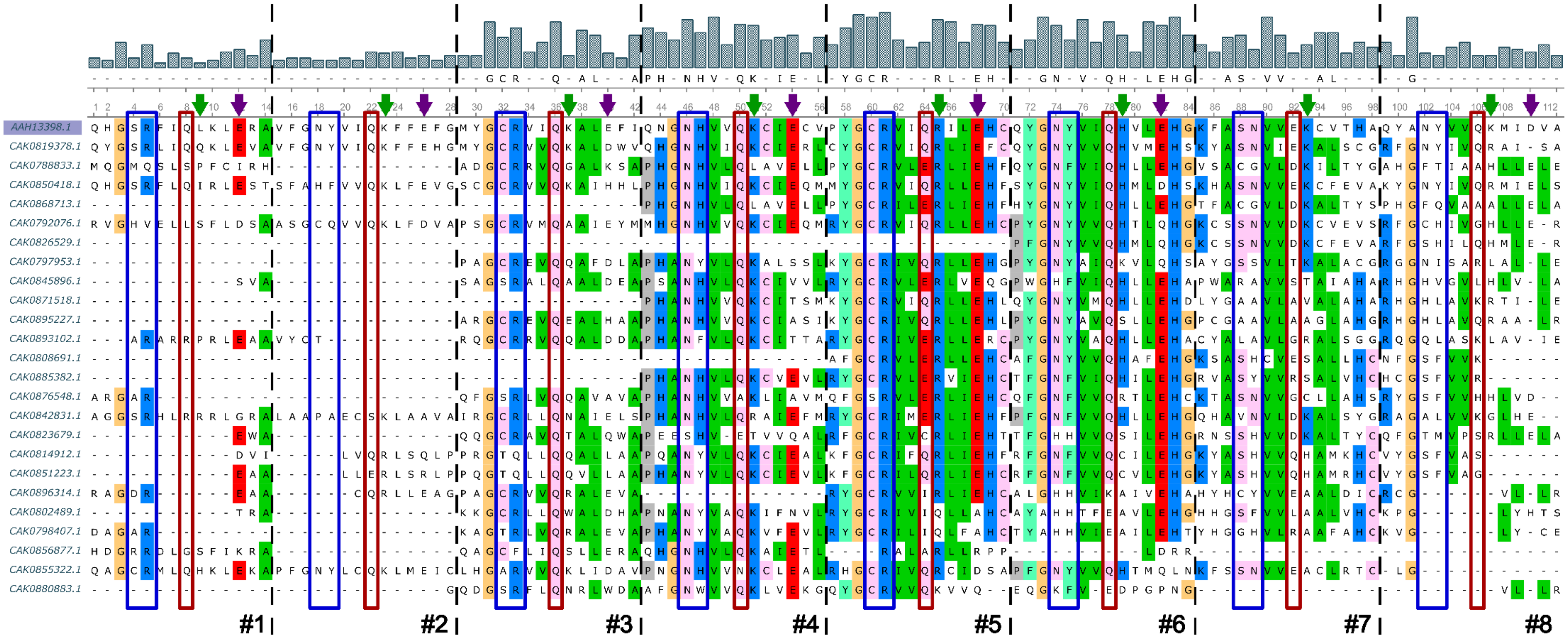

Figure S2. Multiple amino acid sequence alignment of putative Pumilio family proteins from the dinoflagellate *Prorocentrum cordatum* with *Homo sapiens* Pumilio homolog 1 AAH13398.1 (regions containing eight repeated motifs ##1-8). Sequences containing either a Pumilio-family RNA binding repeat (pfam00806) or Pumilio-family RNA binding domain (cd07920) were retrieved from the *P. cordatum* genome assembly GCA_963575745.1 proteome dataset [26]. Residues at positions 12, 13, and 16, corresponding to key RNA-binding residues in characterized Pumilio proteins, are indicated by boxes. Residues at position 16, which form hydrogen bond interactions with RNA, are indicated by brown boxes, whereas residues at positions 12 and 13 indictaed by blue box. Residues at positions 17 and 20, corresponding to residues that can form an electrostatic network together with Glu at position 16 in repeat 7, are marked with green and purple arrows, respectively. Repeat boundaries and positional features were defined in accordance with [171].
