# Supplementary material for: RNA-Binding Proteins in Dinoflagellates
Source: Int J Mol Sci. 2026 Jan 1;27(1):462. doi: 10.3390/ijms27010462 (PMC12787238; doi:10.3390/ijms27010462)
Supplement: Supplementary file 1 [file ijms-27-00462-s001.zip › Figure S3.pdf]

a

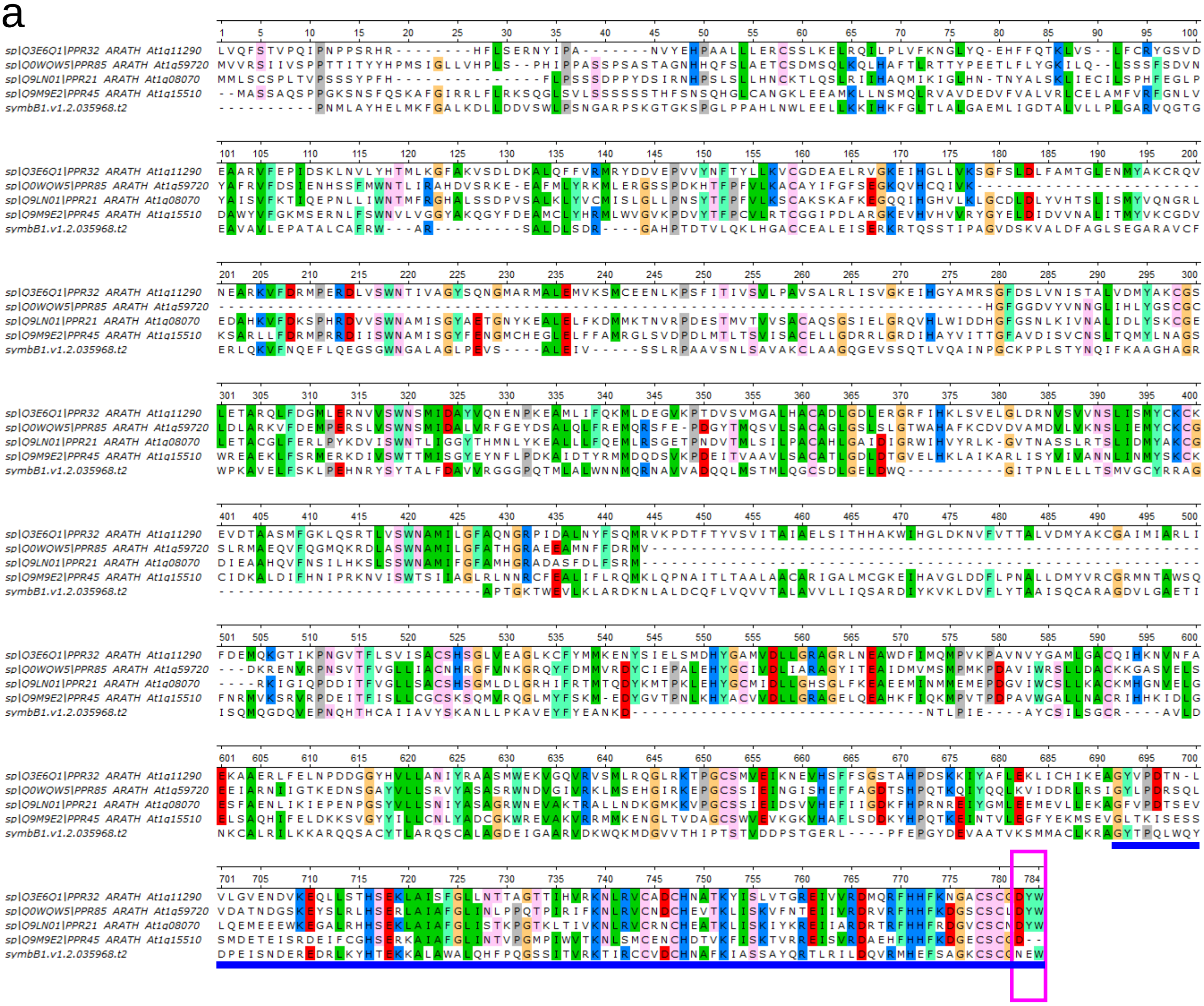
sp|Q3E6Q1|PPR32_ARATH At1g11290
sp|Q0WQW5|PPR85_ARATH At1g59720
sp|Q9LN01|PPR21_ARATH At1g08070
sp|Q9M9E2|PPR45_ARATH At1g15510
symbB1.v1.2.035968.t2

b

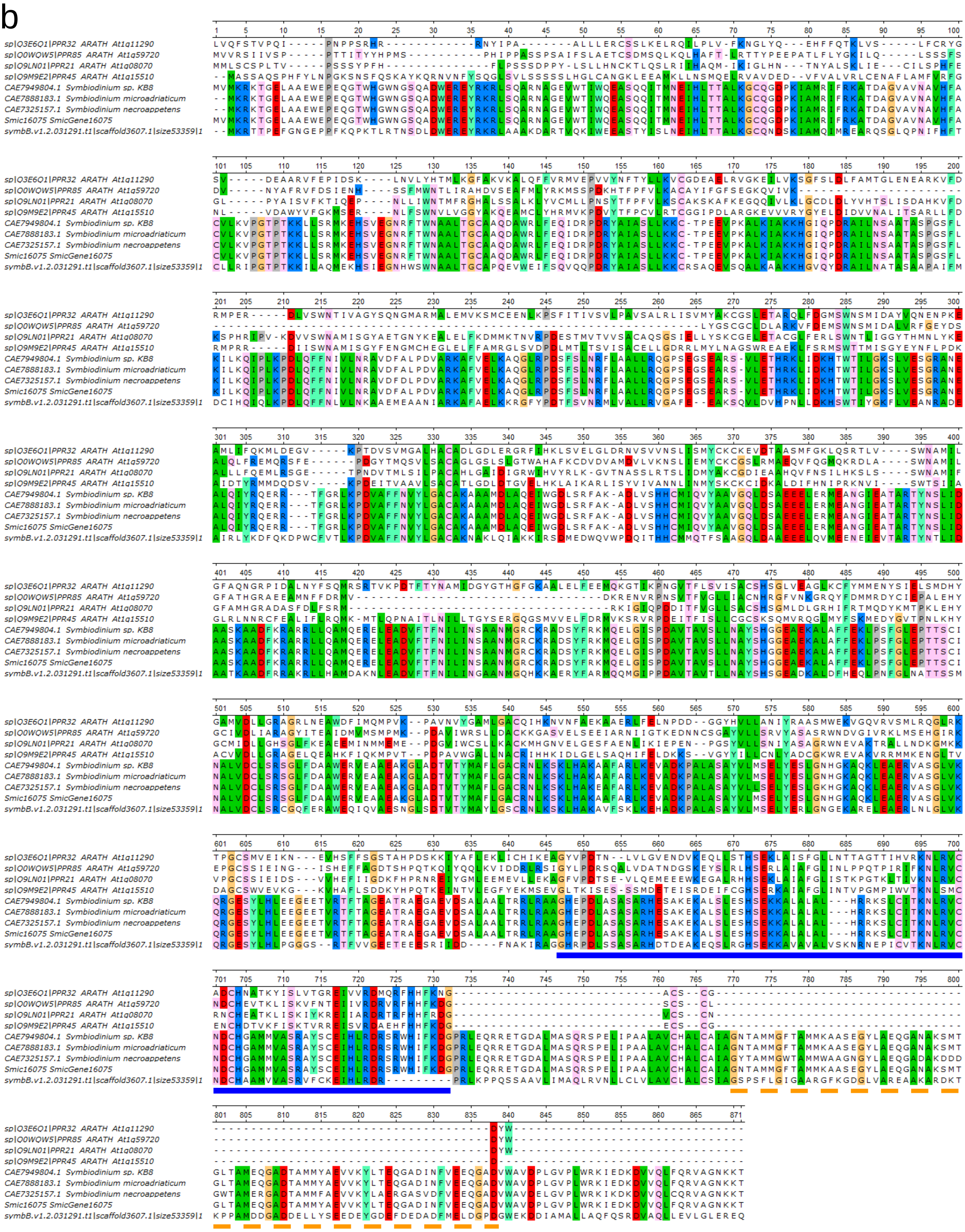

Figure S3. Multiple amino acid sequence alignment of dinoflagellate PPR proteins containing a DYW domain (pfam14432) recognized by the NCBI CD-search tool, compared with *Arabidopsis thaliana* DYW-PPR proteins. **(a)** Multiple sequence alignment including the *Breviolium minutum* sequence symbB1.v1.2.035968.t2, which contains the Asn-Glu-Trp (NEW) residues within the predicted DYW

domain (*blue underline*) at the position corresponding to the canonical Asp-Tyr-Trp (DYW) motif (*pink box*). The C-terminal 159 residues of symbB1.v1.2.035968.t2 , which do not contain conserved domains and could not be aligned, were removed. **(b)** Multiple sequence alignment including *Symbiodinium microadriaticum* and *B. minutum* sequences retrieved from GenBank, ReefGenomics (http://reefgenomics.org/), and the OIST Marine Genomics Unit (https://marinegenomics.oist.jp) databases. These sequences lack well-conserved residues within the predicted DYW domains (*blue underline*) at the position corresponding to DYW motif. Their downstream regions contain ankyrin repeats (*orange dashed underline*; some positions were trimmed). Multiple sequence alignments were generated using MAFFT7 implemented in the Unipro UGENE software package; alignment positions with more than 50% gaps were removed.
