# Supplementary material for: RNA-Binding Proteins in Dinoflagellates
Source: Int J Mol Sci. 2026 Jan 1;27(1):462. doi: 10.3390/ijms27010462 (PMC12787238; doi:10.3390/ijms27010462)
Supplement: Supplementary file 1 [file ijms-27-00462-s001.zip › Table S1.pdf]

Table S1. References to external datasets and supplementary materials reporting RNA-binding protein/domain–related data in dinoflagellates used in the review.

Protein group	References	Referenced information	Dinoflagellate species	Referenced supplementary data
RRM domain-containing proteins	[31]	the loss of RRM1 and RRM2 occurred independently in different lineages	Summarized data	Supplementary material, Table S1
	[6]	a large number of MEI2-like genes with altered expression	*Prorocentrum shikokuense*, *Karenia mikimotoi*, *Scrippsiella acuminata*	Supplementary Material, Figure S2
PUF proteins	[50]	The expression of a gene encoding Pumilio homologue 5 increased approximately 2.5-fold under heat shock conditions	*Symbiodinium microadriaticum*	Additional file 6, dataset “36 °C”
CSD-containing proteins	[26]	57 protein-coding sequences were annotated as “CSP” or “CSP-like” proteins.	*Prorocentrum cordatum*	Additional file 3, Supplementary Table S3
	[32]	Moreover, genes encoding CSD-containing proteins in dinoflagellates frequently occur in tandem arrays	*Fugacium kawagutii*	Supplementary Material, Table S6
	[33]		*Polarella glacialis*	Additional file 3, Supplementary Table 16
	[26]		*Prorocentrum cordatum*	Additional file 3, Supplementary Table S6
DEAD/DEAH-box helicases	[32]	Besides, some genes coding for DEAD-box helicases are listed among those organized into tandem arrays	*Fugacium kawagutii*	Supplementary Material, Table S6
	[33]		*Polarella glacialis*	Additional file 3, Supplementary Table 16
	[26]		*Prorocentrum cordatum*	Additional file 3, Supplementary Table S6
YTH domain family proteins	[26]	Two genes encoding proteins with YTH have been annotated in the *P. cordatum* genome	*Prorocentrum cordatum*	Additional file 3, Supplementary Table S3
YTH domain family proteins	[45]	Mentions of YTH domains in dinoflagellates	aggregated dataset	Supplementary Tables, Table S6

		are also found in Roy et al. (Roy et al., 2018) and Stephens et al. (2018)		
PPR proteins	[32]	Genomic evidence indicates PPR genes are definitely among the genes arranged into blocks of tandemly duplicated genes	*Fugacium kawagutii*	Supplementary Material, Table S6
	[33]		*Polarella glacialis*	Additional file 3, Supplementary Table 16
	[26]		*Prorocentrum cordatum*	Additional file 3, Supplementary Table S6
	[72]	For *B. minutum*, the number of annotated P-class PPR-coding genes varies from 493 (Aranda et al., 2016) to 620 (Mungpakdee et al., 2014), or even 687 (per the main text) / 784 (per the supplementary data) in Cheng et al.	*Breviolum minutum*	Supporting Information, Table S4, dataset “Symbiodinium_minutum”
